# Order vs. Disorder: Cholesterol and Omega-3 Phospholipids Determine Biomembrane Organization

**DOI:** 10.3390/ijms23105322

**Published:** 2022-05-10

**Authors:** Augusta de Santis, Ernesto Scoppola, Maria Francesca Ottaviani, Alexandros Koutsioubas, Lester C. Barnsley, Luigi Paduano, Gerardino D’Errico, Irene Russo Krauss

**Affiliations:** 1 Department of Chemical Sciences, University of Naples Federico II, I-80126 Naples, Italy; augudesantis@gmail.com (A.d.S.); luigi.paduano@unina.it (L.P.); 2 CSGI (Consorzio per lo Sviluppo dei Sistemi a Grande Interfase), I-50019 Florence, Italy; 3 Max Planck Institut für Kolloid und Grenzflächenforschung, 14476 Potsdam, Germany; ernesto.scoppola@mpikg.mpg.de; 4 Department of Pure and Applied Sciences, University of Urbino, I-61029 Urbino, Italy; maria.ottaviani@uniurb.it; 5 Jülich Centre for Neutron Science (JCNS) at Heinz Maier-Leibnitz Zentrum (MLZ), 85748 Garching, Germany; a.koutsioumpas@fz-juelich.de (A.K.); barnslel@ansto.gov.au (L.C.B.); 6 Australian Synchrotron, ANSTO, Clayton 3168, Australia

**Keywords:** cholesterol, phospholipids, omega-3, electron paramagnetic resonance, small angle neutron scattering, neutron reflectivity

## Abstract

Lipid structural diversity strongly affects biomembrane chemico-physical and structural properties in addition to membrane-associated events. At high concentrations, cholesterol increases membrane order and rigidity, while polyunsaturated lipids are reported to increase disorder and flexibility. How these different tendencies balance in composite bilayers is still controversial. In this study, electron paramagnetic resonance spectroscopy, small angle neutron scattering, and neutron reflectivity were used to investigate the structural properties of cholesterol-containing lipid bilayers in the fluid state with increasing amounts of polyunsaturated omega-3 lipids. Either the hybrid 1-stearoyl-2-docosahexaenoyl-sn-glycero-3-phosphocholine or the symmetric 1,2-docosahexaenoyl-sn-glycero-3-phosphocholine were added to the mixture of the naturally abundant 1-palmitoyl-2-oleyl-sn-glycero-3-phosphocholine and cholesterol. Our results indicate that the hybrid and the symmetric omega-3 phospholipids affect the microscopic organization of lipid bilayers differently. Cholesterol does not segregate from polyunsaturated phospholipids and, through interactions with them, is able to suppress the formation of non-lamellar structures induced by the symmetric polyunsaturated lipid. However, this order/disorder balance leads to a bilayer whose structural organization cannot be ascribed to either a liquid ordered or to a canonical liquid disordered phase, in that it displays a very loose packing of the intermediate segments of lipid chains.

## 1. Introduction

Biological membranes are highly complex systems, able to act as a selectively permeable barrier to maintain different conditions and compositions between external and internal compartments [1]. They are formed by a great variety of molecules: lipids, proteins, and carbohydrates, with lipids forming a bilayer that is the scaffold of the whole membrane. Within the bilayer, lipids have several degrees of freedom, such as diffusion along the plane of the membrane, rotation around an axis perpendicular to the membrane plane, fluctuations in and out with respect to the plane of the membrane, etc. [2,3], in addition to fast intra-molecular rotational motions. On these bases, different phases have been identified in biomembranes, including gel and fluid phases, differing for lipid packing and long range order, and, within the fluid phase, the liquid disordered (Ld) and the liquid ordered (Lo) ones, both characterized by a high lateral lipid mobility, but differing in the conformational freedom of the lipid tails [4]. Their stability depends on the lipid composition [5]. However, the great lipid variety in addition to the high number of their degrees of freedom poorly reconcile with a description of the membrane based on just these phases [5,6]. Notably, the existence of domains with different structural and chemico-physical properties within the membrane is increasingly recognized as an important regulatory mechanism for biological processes [5,7,8].

Among the different lipids, polyunsaturated fatty acids (PUFA) and omega-3 fatty acids have attracted a particularly great interest for their many different associated health benefits, including prevention or treatment of neurological diseases [9,10], relief of symptoms of inflammatory disorders [11,12,13], improvements in whole body metabolism [14,15], and prevention of the progress of certain cancers [16,17]. Omega-3 fatty acids were proposed to exert their biological activities through different mechanisms, such as: acting as lipid mediator precursors, transcriptional regulators, modulators of membrane protein functions, and, importantly, by shaping the membranes as either free molecules or components (“apolar tails”) of glycerophospholipids [18,19]. The last point is directly linked to the peculiar chemical structure of omega-3; their long carbon chain with multiple double bonds—for example 20 carbon atoms and 5 double bonds in the case of eicosapentaenoic acid (EPA) and 22 carbon atoms and 6 double bonds in the case of docosahexaenoic acid (DHA), the two most representative members of this family [20]—allows a great degree of conformational flexibility that inevitably affects the physico-chemical and structural properties of the membranes in which they are embedded [21]. However, the mechanisms and significance of omega-3 incorporation in membranes have remained obscure, compared to the other biological roles of these molecules [18]. In this respect, we have recently indicated that small amounts of the di-DHA phospholipid 1,2-docosahexaenoyl-*sn*-glycero-3-phosphocholine (22:6–22:6PC) are able to perturb liquid disordered bilayers by increasing their fluidity slightly but sufficiently to promote morphological rearrangements [22]. It was demonstrated that, beyond a threshold concentration, 22:6–22:6PC impairs formation of lamellar phases in Ld lipid membranes and induces the formation of small spherical aggregates prone to clusterize, as the likely result of the partial exposure of the acyl chains to the aqueous medium [22]. These results prove the ability of omega-3 phospholipids to strongly affect membrane morphology and properties.

A key component of eukaryotic cell membranes is cholesterol, thanks to its ability to control membrane fluidity and organization, in addition to other physicochemical parameters. Cholesterol interaction with phospholipids creates the Lo phase [23]: it increases the order of the fluid phase and decreases the order of the gel phase, resulting in the Lo phase. Cholesterol decouples translational and configurational order, which are both low in the Ld phase and high in the gel phase, such that the Lo phase is characterized by a high configurational order and a low translational one [24]. Cholesterol also plays an essential regulatory function in many biomembrane processes [25], protein and enzyme activities [26,27], and the formation of raft-like domains [28,29,30], in turn associated with cell signalling and intracellular trafficking [31,32]. A great number of studies dealt with cholesterol effects on membrane properties and membrane-associated events and several features of cholesterol-embedding membranes have been deeply understood, for example the condensing effect, or the umbrella interaction with PC lipids [33,34,35,36,37]. Nonetheless, structure–property relationships of this molecule are not yet completely unveiled and unexpected or surprising results could still be obtained when working with systems containing cholesterol, as it is the case, for example, of cholesterol effects on membrane bending rigidity, with a stiffening action exerted on membranes composed by saturated lipids, but not on those formed by unsaturated lipids [38].

Due to the contrasting effects they exert (ordering vs. disordering), the study of systems composed of both cholesterol and polyunsaturated lipids is particularly interesting [39,40,41]. One of the most intriguing aspects of these systems regards the location of cholesterol in the bilayer [23]. In lipid bilayers, cholesterol is generally found intercalated among the phospholipid tails in numerous orientations, most of which correspond to a “canonical” orientation roughly parallel to the bilayer normal, with the polar hydroxyl group close to the surface [42]. In contrast, in the case of membranes embedding polyunsaturated lipids, cholesterol was reported to occupy an unusual midplane location, lying flat between the two lipid leaflets, perpendicular to the bilayer normal [43,44,45]. Such a position has been revised and corrected with years, with several authors now tending towards a tilted rather than flat cholesterol orientation [46]. A crucial role has been proposed to be played by the thickness of the bilayer rather than by the degree of the unsaturation of lipids [47]. Notably, computational studies often failed to model the in-plane cholesterol position [48] unless specific constraints were employed [23,49]. Quite recently, it was proposed that only a very small percentage of cholesterol was indeed able to move from the canonical in-leaflet position to the in-plane one [23,50]. Thus, the cholesterol location in PUFA-enriched lipid bilayer deserves further investigation.

Another open issue concerns the capacity of polyunsaturated phospholipids to segregate with respect to cholesterol-rich domains; phospholipids esterified by a single PUFA together with a saturated chain, thus forming a hybrid lipid, were found in raft extracts [51,52], and were also proven to partition well to cholesterol-rich ordered membrane regions [53], despite their disordered nature. Conversely, di-PUFA phospholipids were generally reported to segregate with respect to cholesterol-enriched bilayers [54]. The combined effects of cholesterol and PUFA were described in terms of a coexistence of Lo and Ld phases [55].

With the aim at clarifying these points, the present study investigates the effect of cholesterol on the behaviour of two classes of phospholipids containing the longest and most unsaturated omega-3 acyl chain, that is docosahexaenoic acid (DHA): (a) a hybrid phospholipid containing DHA and a saturated chain, 1-stearoyl-2-docosahexaenoyl-*sn*-glycero-3-phosphocholine (18:0–22:6PC), and (b) the symmetric di-DHA phospholipid 1-2-docosahexaenoyl-*sn*-glycero-3-phosphocholine (22:6–22:6PC). As the third component of the lipid mixtures including cholesterol and either 18:0–22:6PC or 22:6–22:6PC, we chose the naturally abundant 1-palmitoyl-2-oleyl-*sn*-glycero-3-phosphocholine (POPC) (Figure S1). Although these simple lipid mixtures cannot be considered highly biomimetic, they may serve as simplified model systems allowing a general understanding of the behaviour of each lipid and how their combination determines the overall membrane properties, overriding problems arising from the high compositional complexity of plasma membranes in addition to their variability from one kind of cell to the other [50].

Membranes in a large range of lipid compositions (as detailed in Table 1) were investigated by means of electron paramagnetic resonance spectroscopy (EPR) to characterize the microstructural features of the lipid arrangement, while morphological features of selected representative samples were investigated by means of neutron reflectivity (NR) and small angle neutron scattering (SANS).

## 2. Results

### 2.1. Composition of Lipid Mixtures

Our study focuses on membranes formed by ternary lipid mixtures including chol, one polyunsaturated lipid (either 18:0–22:6PC or 22:6–22:6PC) and POPC. In each system, the sum of the polyunsaturated lipid and POPC constitutes the total phosphocholine (PC) content. For chol concentration, since its percentage in plasma membranes is about 40 mol % of total lipids [25], and the shortcomings of conducting experiments and simulations at about 20 mol % concentrations have been proven [23], we selected a fixed cholesterol content of 40 mol % of total lipids in all the investigated systems (i.e., 4:6 chol:PC molar ratio). Indeed, up to 25–30% cholesterol content, the Lo phase coexists with Ld and gel phases, while above this threshold only Lo is present [56]. Conversely, the following whole range of polyunsaturated lipid fraction over the total PC was investigated:

$$0\leq x_{18:0–22:6\mathrm{PC}}=\frac{{\mathrm{mol}}_{18:0–22:6\mathrm{PC}}}{{\mathrm{mol}}_{\mathrm{PC}}}=\frac{{\mathrm{mol}}_{18:0–22:6\mathrm{PC}}}{{\mathrm{mol}}_{18:0–22:6\mathrm{PC}}+{\mathrm{mol}}_{\mathrm{POPC}}}\leq 1$$

$$0\leq x_{22:6–22:6\mathrm{PC}}=\frac{{\mathrm{mol}}_{22:6–22:6\mathrm{PC}}}{{\mathrm{mol}}_{\mathrm{PC}}}=\frac{{\mathrm{mol}}_{22:6–22:6\mathrm{PC}}}{{\mathrm{mol}}_{22:6–22:6\mathrm{PC}}+{\mathrm{mol}}_{\mathrm{POPC}}}\leq 1$$

When directly comparing the effects of the two different polyunsaturated lipids, 18:0–22:6PC and 22:6–22:6PC, at the same content of DHA, the following DHA molar fraction with respect to total phosphocholine tails (PC tails) was employed:

xDHA=molDHAmolPC tails=mol22:6–22:6PCmol22:6–22:6PC+molPOPC=12 mol18:0–22:6PCmol18:0–22:6PC+molPOPC

Explicit compositions of all analysed sample are reported in Table 1. 

### 2.2. EPR Results: Microstructural Features of Vesicles Containing Omega-3 Phospholipids

Insights into the microstructural features of chol/POPC/18:0–22:6PC and chol/POPC/22:6–22:6PC systems were obtained by means of EPR spectroscopy with the spin-probe method. The systems were analysed at a constant cholesterol molar fraction of 0.4 with respect to total lipids, while the phospholipid molar composition was varied in the whole range (0 ≤ x_18:0–22:6PC_ ≤ 1 and 0 ≤ x_22:6–22:6PC_ ≤ 1), as summarized in Table 1. In particular, we employed the following as spin probes: (i) 5-PCSL, to investigate the structure in the outer region of the bilayer just below the polar heads; (ii) 14-PCSL, to investigate the features of the bilayer inner core; (iii) CNO, to specifically investigate the behaviour of cholesterol embedded in bilayers containing polyunsaturated lipids (molecular structures of spin probes are reported in Figure S2). Indeed, CNO was proven to be the best probe to mimic cholesterol behaviour among different cholesterol analogues [57].

All the experimental spectra (Figures S3–S5, Supporting Material) were analysed by computer simulation of the EPR lineshape (examples in Figures S6–S8, Supporting Material). The computation approach was used previously [58,59] for similar systems [22] and allowed us to extract the following main parameters: (a) the hyperfine coupling constant <A>, which is related to the polarity experienced by the paramagnetic label; (b) the correlation time for the label rotational motion τ, which is related to the local microviscosity; (c) the order parameter S, a measure of the orientational ordering of the labelled segment of the acyl chain with respect to the normal bilayer surface. For no spin-label and at no lipid mixture composition, we observed the superposition of EPR signals, which could have been indicative of a spin-label partitioning in two different aggregate types. 

The same g-tensor components (g_ii_ = 2.0075, 2006, 2.003) were found to satisfactorily simulate 5-PCSL and 14-PCSL. As for the CNO, the g_ii_ values (2.014, 2.006, 2.0015) indicate a higher anisotropy of the magnetic moment for CNO with respect to PCSLs, likely due to the rigid polycyclic structure of the molecule able to also affect the NO spectral features. For all the spin-probes, and for increasing the reliability of the most informative parameters, the g-tensors were taken constant by changing the bilayer lipid composition.

The hyperfine coupling constant, similar to the g tensor, was considered constant over the whole phospholipid composition range for both chol/POPC/22:6–22:6PC and chol/POPC/18:0–22:6PC systems, being *<*A*> =* 14.7 G and 14.0 G, for 5-PCSL and 14-PCSL spectra, respectively. The constant <A> values could be considered an oversimplification, but the small change from 14.7 to 14.0 supports the finding that the polarity changes poorly at the probe site. However, the higher <A> value for 5-PCSL reflects a higher local polarity, confirming the correct insertion of the labelled acyl chain in the lipid bilayer, in which the polarity decreases moving from the interface to the inner core. As for CNO, a <A> value of 14.3 G was obtained in the computation, which is between those of 5-PCSL and 14-PCSL, suggesting the NO group of this spin-probe to be positioned at an intermediate depth. 

In contrast to the spectral parameters discussed above, significant variations of the correlation time for motion τ_perp_ (hereafter simply τ), and particularly of the order parameter S as a function of lipid mixture composition were highlighted by simulation of 5-PCSL, 14-PCSL, and CNO spectra for both chol/POPC/22:6–22:6PC and chol/POPC/18:0–22:6PC systems. These parameters are reported in Table S1 and will be separately analysed in the following subsections for the two systems.

#### 2.2.1. Chol/POPC/22:6–22:6PC Lipid Bilayers

**Figure 1:**
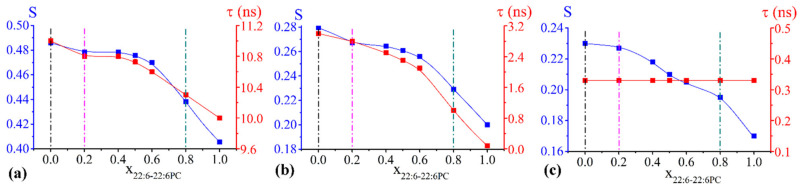
Order parameter S (blue) and correlation time τ (red) of 5-PCSL (**a**), 14-PCSL (**b**), and CNO (**c**) included in chol/POPC/22:6–22:6PC lipid mixtures as a function of x_22:6–22:6PC_. Since errors are within 1%, they may not be clearly visible in the figure. Lipid mixtures that will be further analysed are highlighted by vertical dashed lines.

Concerning the chol/POPC/22:6–22:6PC system, τ and S values obtained from 5-PCSL, 14-PCSL, and CNO spectra simulations are reported as a function of x_22:6–22:6PC_ in Figure 1a–c, respectively.

In the case of 5-PCSL (Figure 1a), both τ and S change slightly for 0.2 < x_22:6–22:6PC_ < 0.6, while above this threshold, an abrupt decrease is observed, indicating that an increase of the di-polyene phosphocholine causes a significant disorder and increased fluidity in the outer region of the bilayer. 

A very similar trend is evident for both τ and S in the case of 14-PCSL, indicating a slight initial decrease of both parameters, a plateau region, and an abrupt decrease at high 22:6–22:6PC concentrations. The much lower values of τ and S obtained for 14-PCSL with respect to 5-PCSL indicate higher disorder and mobility of acyl tails in the inner region of the bilayer than in the outer one. The mobility for 14-PCSL is much higher than that measured for 5-PCSL, as expected for the different nitroxide group location, but it is lower than what we measured when handling similar membrane systems but devoid of cholesterol and therefore adopting an Ld phase [22]. Incidentally, the relative low mobility of 14-PCSL can be related to the high conformational order of the acyl phospholipid chains that, in the Lo phase, is similar to the gel phase, while the two phases differ for other features such as lateral mobility of the phospholipids, long-range order, and bilayer hydration [24,60,61].

We underline here that τ and S follow similar trends, but the computational procedure was less sensitive to τ variations. In this respect, CNO spectra were computed with the same probe rotational mobility with the addition of 22:6–22:6PC, being τ = 0.33 ns over the whole composition range. Such a value for correlation time, significantly smaller than those found for 5- and 14-PCSL, indicates a faster mobility of CNO if compared to the PC probe that can be related to its smaller molecular size (Figure S2). Conversely, the S parameter decreases with increasing x_22:6–22:6PC_, the largest variation being observed above x_22:6–22:6PC_ = 0.8 (Figure 1c).

Overall, the addition of 22:6–22:6PC increases the disorder of the whole bilayer, from the outer portion to the inner core, with the most evident changes observed at high omega-3 phospholipid content (i.e., beyond the x_22:6–22:6PC_ = 0.6 threshold, at least).

#### 2.2.2. Chol/POPC/18:0–22:6PC Lipid Bilayers

**Figure 2:**
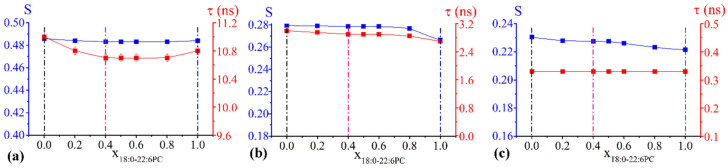
Order parameter S (blue) and correlation time τ (red) of 5-PCSL (**a**), 14-PCSL (**b**), and CNO (**c**) included in chol/POPC/18:0–22:6PC lipid mixtures as a function of x_18:0–22:6PC_. Since errors are within 1%, they may not be clearly visible in the figure. Lipid mixtures that will be further analysed are highlighted by vertical dash lines.

In the case of chol/POPC/18:0–22:6PC, the variations of τ and S observed with the three spin probes (Figure 2a–c) are much smaller than those found for chol/POPC/22:6–22:6PC (see also Figure S9, Supporting Material). Nonetheless, they are above the accuracy of the measure. 

In the case of 5-PCSL (Figure 2a), both τ and S decrease up to x_18:0–22:6PC_ = 0.4, remain constant up to x_18:0–22:6PC_ = 0.8 and slightly increase at higher chol/18:0–22:6PC content, with values matching those obtained at x_18:0–22:6PC_ = 0.2. In the case of 14-PCSL (Figure 2b) and CNO (Figure 2c) the changes of τ and S resemble those observed for the chol/POPC/22:6–22:6PC system, but much less marked. For 14-PCSL, the sequence initial decrease, plateau region, and abrupt decrease is observed for both parameters. This sequence is also observed for the CNO S, while τ remains constant over the whole composition range.

#### 2.2.3. S Profiles

For selected lipid mixtures, highlighted in Figure 1 and Figure 2 by vertical dashed lines, we decided to delve deeper into the characterization of the systems at the microscopic/molecular level. For this reason, the entire profile of the local structuring experienced by the lipid acyl chain segments was determined by adding two additional probes, namely 7-PCSL and 10-PCSL, to the spin-probe analysis. Lipid compositions to be scrutinized where chosen as follows:

- concerning the chol/POPC/22:6–22:6PC system, we considered two lipid compositions: one well below (x_22:6–22:6PC_ = 0.2) and the other above (x_22:6–22:6PC_ = 0.8) the threshold (x_22:6–22:6PC_ = 0.6) at which a dramatic change of the spectral features was observed (Figure 1);
- concerning the chol/POPC/18:0–22:6PC system, we chose the lipid mixtures with a DHA content as close as possible to those of chol/POPC/22:6–22:6PC systems, i.e., x_18:0–22:6PC_ = 0.4 and x_18:0–22:6PC_ = 1 (the last corresponding to chol/18:0–22:6PC system), in order to allow a direct analysis of the effects of PUFA molecular distribution (i.e., monopolyenes vs. dipolyenes)

Last, we considered the chol/POPC lipid mixture for comparison.

**Figure 3:**
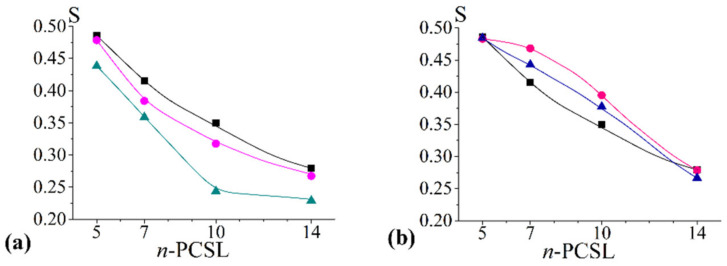
Order parameter S as a function of the spin label *n*-position in *n*-PCSLs, for (**a**) chol/POPC (black), chol/POPC/22:6–22:6PC x_22:6–22:6PC_ = 0.2 (magenta) and 0.8 (cyan); (**b**) chol/POPC (black), chol/POPC/18:0–22:6PC x_18:0–22:6PC_ = 0.4 (pink) and 1 (blue). Since errors are within 1%, they may not be clearly visible in the figure.

The S profiles for the selected chol/POPC/22:6–22:6PC and chol/POPC/18:0–22:6PC lipid mixtures as a function of the nitroxide-label position in *n*-PCSLs are displayed in Figure 3a,b, respectively. τ parameters are not reported since they indicated similar trends as S variations, but τ variations were relatively small.

In the case of chol/POPC, the S parameter decreases gradually from 5- to 14-PCSL (Figure 3a,b, black squares). The S profile for chol/POPC/22:6–22:6PC x_22:6–22:6PC_ = 0.2 is quite similar to that of chol/POPC, with a slight decrease of the order observed at all bilayer depths. Conversely, in the case of chol/POPC/22:6–22:6PC at x_22:6–22:6PC_ = 0.8, the S profile is different: for 10- and 14-PCSL, the S parameter assumes a very low and constant value, which indicates a dramatically increased disorder related to a higher mobility allowed by the bending of the polyunsaturated tails of 22:6–22-6PC. However, only a modest decrease is observed for 5- and 7-PCSL.

As for the chol/POPC/18:0–22:6PC system, a completely different behaviour is observed: in the presence of the hybrid omega-3 phosphocholine, a significant increase of order with respect to chol/POPC bilayers is found in the intermediate region of the bilayer, as probed by 7- and 10-PCSL. The order in this region is maximum at x_18:0–22:6PC_ = 0.4 and slightly decreases, increasing 18:0–22:6PC content. Conversely, the presence of 18:0–22:6PC minimally affects order at the other label positions, i.e., close to the lipid headgroup and at the tail termini.

These findings highlight a very different effect of hybrid and symmetric omega-3 phosphocholines on the microstructural features of the lipid bilayer. This is evident when comparing lipid mixtures with the same x_DHA_ = 0.2 (i.e., x_22:6–22:6PC_ = 0.2 and x_18:0–22:6PC_ = 0.4), the former indicating a decrease and the latter an increase of order by a similar extent. With increasing the PUFA content, chol/POPC/18:0–22:6PC lipid mixtures remain poorly affected, while chol/POPC/22:6–22:6PC mixtures indicate a dramatic decrease of the lipid tail ordering. 

### 2.3. SANS Results: Morphology of chol/POPC/22:6–22:6PC Aggregates

With the aim of verifying whether the decreased tail ordering in 22:6–22:6PC enriched bilayers is able to induce structural changes on a larger scale, as previously observed in systems devoid of cholesterol [22], SANS experiments were performed, in particular with x_22:6–22:6PC_ = 0.2 and 0.8 and chol/POPC mixtures for comparison.

**Figure 4:**
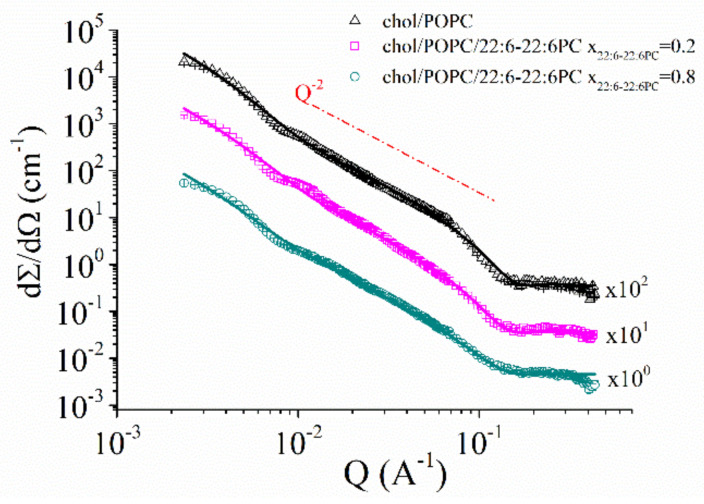
SANS experimental points and best fitting curves for chol/POPC (black), chol/POPC/22:6–22:6PC x_22:6–22:6PC_ = 0.2 (magenta), 0.8 (cyan). The Q^−2^ slope is explicitly illustrated as a dashed line. For the sake of clarity, the SANS profiles were shifted by multiplication as indicated in the figure.

Analysis of Figure 4 highlights that all SANS profiles are very similar: (i) no peak is present in all cases, and the absence of a peak at high-q indicates that the lipid aggregates do not have a repetitive multilamellar structure with a fixed distance among stacked lamellae. Conversely, the absence of a peak at low-Q indicates that there is no interaggregate interaction; (ii) at intermediate q values, the profiles follow a power law $d\Sigma /d\Omega \propto Q^{-\alpha }$ with α slightly higher than 2, indicative of the presence of some multilamellar structures.

A detailed quantitative analysis was performed by fitting the SANS data using the SASView program [62]. In all cases, no structure factor was considered. For what concerns the form factor, a lamellar stack paracrystal model [63] was the one giving the best results among the different models tested. This model is usually employed for the treatment of large multilamellar vesicles. Structural parameters as derived from fitting of SANS data are reported in Table 2.

Data in Table 2 indicate, for all the systems, a limited multilamellarity; by increasing the 22:6–22:6PC content, a slight decrease of lamellae number occurs. Conversely, thickness remains unvaried with respect to chol/POPC in the case of chol/POPC/22:6–22:6PC at x_22:6–22:6PC_ = 0.2 and slightly decreases at higher omega-3 concentrations. No dramatic change or phase transition can be inferred based on these data, thus confirming the lamellar structure of all the considered lipid mixtures.

### 2.4. NR Results: Mesostructure of Supported Lipid Bilayers Containing Omega-3 Phospholipids

With the aim of investigating mesoscopic features of bilayers composed of POPC, cholesterol, and either the hybrid 18:0–22:6PC or the symmetric di-DHA phospholipid 22:6–22:6PC, we performed neutron reflectivity experiments on selected samples representative of lipid mixture with a small content of omega-3, a high content of omega-3, and on a chol/POPC system for comparison. We selected the systems with x_22:6–22:6PC_ = 0.2 and 0.8 and with x_18:0–22:6PC_ = 0.4 and 1, as already done in the case of the determination of the S profile (see above). All the samples were analysed in three different contrast media: D_2_O, H_2_O, and silicon-matched water (SiMW).

NR profiles were analysed by using a fitting procedure that employs as input parameters the scattering length densities and molecular volumes of all the chemical components (Table S2), which are: (1) the silicon support, (2) the silicon oxide layer on which the lipid bilayer is deposited, (3) the thin water layer interposed between the support and the lipid bilayer, (4) the lipid headgroups, and (5) the lipid tails, considering the different lipid compositions. 

The fitting is based on parameterized volume fraction profiles of all these components, while the lipid bilayers are modelled as three slabs: two identical slabs corresponding to the headgroup regions sandwiching one single slab corresponding to the tail region. For each slab, the thickness, scattering length density (*SLD*), solvent volume fraction, and interfacial roughness were obtained by fitting the experimental NR curves, thus furnishing information about the structural organization of the supported membrane. To describe the z profile distribution of all chemical components in the system, the modelling process was performed in several steps. At first, a roughness-free volume fraction distribution is generated. Subsequently, all chemical components are convolved by a Gaussian function of width equal to their intrinsic roughness (Bilayer Roughness in Table 3). The final volume fraction profile is obtained by an additional convolution by a Gaussian function of width equal to the substrate roughness and then translated to the *SLD* profile generating the model reflectivity R(Q). This procedure was proved to reveal the volume fraction distribution model behind the observed *SLD* profile [22,64,65].

#### 2.4.1. Chol/POPC/22:6–22:6PC

**Figure 5:**
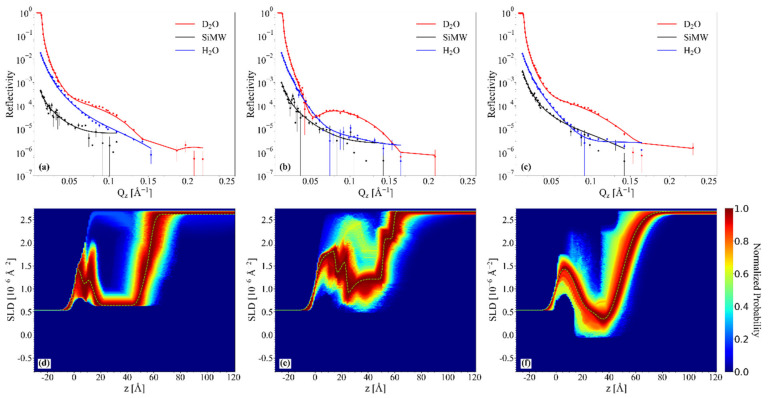
Neutron reflectivity results for chol/POPC (**a**,**d**), chol/POPC/22:6–22:6PC with x_22:6–22:6PC_ = 0.2 (**b**,**e**) and 0.8 (**c**,**f**): experimental data and best fitting curves in D_2_O (red), silicon match water (black), and H_2_O (blue) (panels (**a**–**c**)); *SLD* probability maps in D_2_O (panels (**d**–**f**)).

In Figure 5, the NR profiles of chol/POPC (a), chol/POPC/22:6–22:6PC with x_22:6–22:6PC_ = 0.2 (b), and 0.8 (c) in the three contrast media are displayed, reporting both experimental data and best fitting curves. Data fitting is satisfactory in each contrast media (see also Fresnel representation ${\mathrm{RQ}}_{z}^{4}\;\mathrm{vs}.{\;Q}_{z}$ of experimental data and best fitting curves in Figure S10a–c, Supplementary Material). NR profiles of different lipid systems are similar from one system to the other.

Since we previously observed the detachment of lipids from the support induced by a massive structural organization in POPC/22:6–22:6PC systems with high content of 22:6–22:6PC [22], in no way were the simulations bound to impose the presence of a supported lipid bilayer. As can be clearly seen from *SLD* probability maps (Figure 5d–f), in all the cases the highest probability corresponds to an *SLD* profile with a deep well at about 30–40 Å, as it is found for model supported lipid bilayers [22,66]. *SLD* profiles in different contrast media are reported in Figure S11a–c (Supporting Material).

Analysis of structural parameters derived from fitting (Table 3) allows a thorough description of the 22:6–22:6PC effects on the lipid bilayers. Chol/POPC bilayer is the thickest one, with a tail thickness of about 36 Å, significantly higher than that of pure POPC bilayers [22], and the more packed, with an area/lipid value of about 45 Å^2^ [22]. These findings perfectly agree with the known ability of cholesterol to induce an increase of thickness and lipid packing [37].

The addition of 22:6–22:6PC causes a thinning of the bilayer as the omega-3 concentration increases. In particular, the tail thickness of 23 Å observed at x_22:6–22:6PC_ = 0.8 points toward a significant folding back of polyunsaturated tails. At the same time, headgroup thickness increases with 22:6–22:6PC concentration. These two results seem to poorly reconcile with the hypothesized in-plane position of cholesterol, and rather point to a perturbation of cholesterol–phosphocholine interaction caused by DHA tails folding back. Moreover, at high 22:6–22:6PC concentration, the bilayer roughness is higher than in other systems, and this finding could be also related to the partial bending of unsaturated tails, or to compositional and/or phase inhomogeneity [67]. The area/lipid values, although higher than that of chol/POPC, nonetheless indicate a high coverage of the support. 

**Figure 6:**
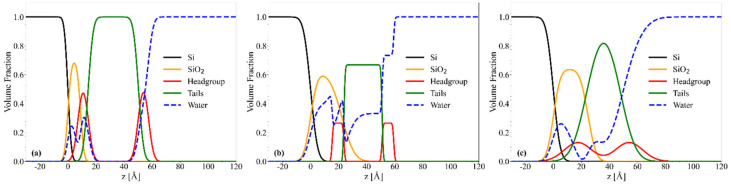
Convolved volume fraction distribution profiles for chol/POPC (**a**), chol/POPC/22:6–22:6PC with x_22:6–22:6PC_ = 0.2 (**b**) and 0.8 (**c**).

These features emerge from inspection of convolved volume fraction distribution profiles (Figure 6) (corresponding not-convolved volume fraction distribution profiles are reported in Figure S12), too. Incidentally, smoothness and overlap of convolved volume fraction distribution profiles are apparent effects of the lipid bilayer and silicon substrate roughness. It should be noted that at low 22:6–22:6PC concentration, there are a higher area/lipid value and a not negligible water content in the tail region (Figure 6b) which could be related to stiffer starting vesicles that fuse slightly worse on the substrates than those at x_22:6–22:6PC_ = 0.8. 

#### 2.4.2. Chol/POPC/18:0–22:6PC

**Figure 7:**
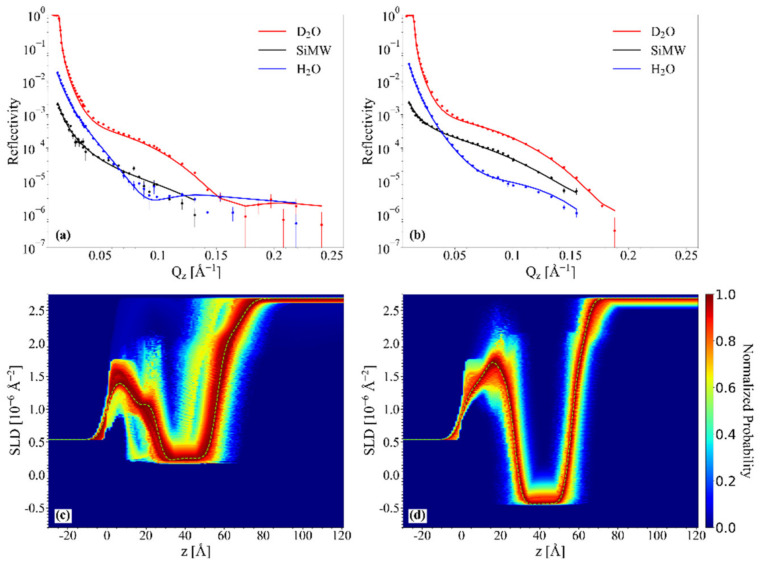
Neutron reflectivity results for chol/POPC/18:0–22:6PC with x_18:0–22:6PC_ = 0.4 (**a**,**c**) and chol/18:0–22:6PC (**b**,**d**): experimental data and best fitting curves in D_2_O (red), silicon match water (black), and H_2_O (blue) (panels (**a**,**b**)); *SLD* probability maps in D_2_O (panels (**c**,**d**)).

Good quality fits are also obtained for chol/POPC/18:0–22:6PC systems, as can be seen in Figure 7a,b and Figure S10d,e (Supporting Material). Even for this system, *SLD* probability maps indicate the presence of supported lipid bilayers, with a deep well at about 40 Å. *SLD* profiles in different contrast media are reported in Figure S11d,e (Supporting Material).

**Figure 8:**
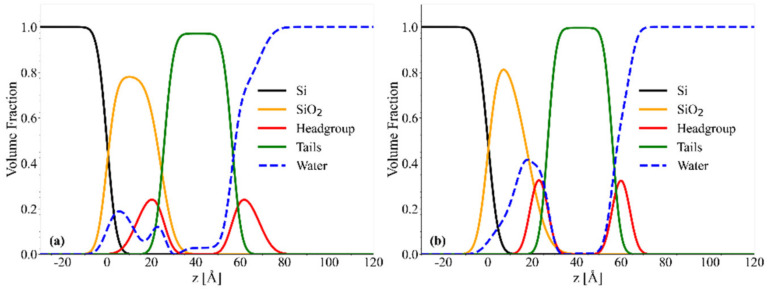
Convolved volume fraction distribution profiles for chol/POPC/18:0–22:6PC with x_18:0–22:6PC_ = 0.4 (**a**), and chol/18:0–22:6PC (**b**).

Structural parameters, reported in Table 3, and convolved volume fraction distribution profiles (Figure 8) indicate that the addition of 18:0–22:6PC induces a thinning of the bilayer and an increase of area/lipid with respect to chol/POPC. This behaviour is similar to that of the di-DHA phospholipid, but the extent of these changes is lower for 18:0–22:6PC than for 22:6–22:6PC. In particular, the tail thickness decreases from 36 Å to about 30 Å in the presence of 18:0–22:6PC, independent of the omega-3 concentration; the presence of the saturated tail impairs the formation of a very thin bilayer even in the case of chol/18:0–22:6PC system. Conversely, the area/lipid is higher for chol/POPC/18:0–22:6PC x_18:0–22:6PC_ = 0.4 than for chol/18:0–22:6PC, suggesting a better packing of the latter bilayer. In all cases, a high support coverage can be inferred from area/lipid values. 

Finally, the comparison of convolved volume fraction distribution profiles of two systems with x_DHA_ = 0.2 (Figure 6b and Figure 8a) indicates that the water content in the tail region is much lower and overall negligible in chol/POPC/18:0–22:6PC systems (Figure 8a) with respect to 22:6–22:6PC containing ones (Figure 6b), despite presenting the same DHA content.

## 3. Discussion

In this work, we have investigated in detail the effect of omega-3 phospholipids, particularly the hybrid 18:0–22:6PC and the symmetric 22:6–22:6PC, on model lipid membranes in the liquid ordered phase containing 40% mol/mol cholesterol. The phosphocholine content was varied, as detailed in Table 1. First, the self-structuring and dynamics of the lipid molecules constituting these membranes were studied as a function of the increasing polyunsaturated phospholipid concentration by employing EPR spectroscopy with the spin probe approach; second, the morphology of the aggregates was checked by means of SANS experiments; lastly, the mesoscopic structure of the bilayer was investigated by NR.

An integrated analysis of the results described in previous sections allowed a wealth of considerations on the behaviour of lipid membranes in which cholesterol and omega-3 phospholipids are both present, as detailed below.

### 3.1. The Nature of the Polyunsaturated Lipid, Rather than the Overall DHA Content, Defines the Lipid Membrane Organization

Characterization of lipid bilayers by NR indicates that 18:0–22:6PC and 22:6–22:6PC affect mesoscopic features in a similar way, even if to a different extent. Indeed, the addition of omega-3 phospholipids reduces the packing of lipids and causes a thinning of the tail region of the bilayer, as verified by SANS as well. Such a finding can be related to the conformational variability of polyunsaturated tails that are able to fold back thus reducing the bilayer thickness and increasing the area/lipid. However, the thinning effect is much lower in the case of 18:0–22:6PC, indicating that the presence of the saturated chain impairs the dramatic thinning that is observed with 22:6–22:6PC.

Conversely, results relating to the microscopic features obtained by means of EPR indicate that 22:6–22:6PC and 18:0–22:6PC affect lipid membranes differently; the phospholipid bearing two polyunsaturated tails increases disorder of the whole lipid tail segments, and the hybrid with a single omega-3 tail slightly increases lipid order of the bilayer intermediate region, as indicated by analysis of S profiles, while barely affecting order in the outer and inner regions (Figure 3b).

**Figure 9:**
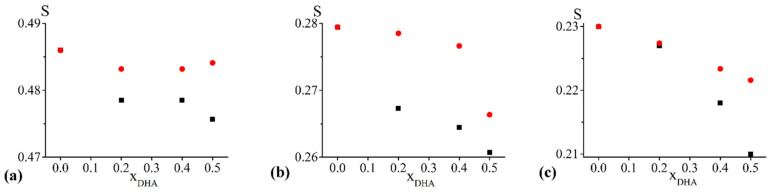
Comparison between the S parameter of chol/POPC/22:6–22:6PC (black) and chol/POPC/18:0–22:6PC (red) at the same DHA content (see Table 1 for definitions). Results obtained with 5-PCSL (**a**), 14-PCSL (**b**), and CNO (**c**). Since errors are within 1%, they may not be clearly visible in the figure.

Notably, these differences between the effects of the two omega-3 phospholipids cannot be ascribed to the different overall content of the polyunsaturated DHA, but rather to the nature of the phospholipid itself. This appears evident if we compare systems containing either 22:6–22:6PC or 18:0–22:6PC with the same content of DHA. To clarify this point, Figure 9 compares systems containing either 22:6–22:6PC or 18:0–22:6PC with the same content of DHA. In Figure 9 we report the S data displayed in Figure 1 (for 22:6–22:6PC) and Figure 2 (for 18:0–22:6PC) as a function of x_DHA_ (see Table 1). All the spin-probes indicate that lipid bilayers containing 18:0–22:6PC are more ordered than those containing 22:6–22:6PC.

This evidence could be related to the different attitude of cholesterol to interact with the two omega-3 phospholipids. The rigid steroidal polycyclic structure has higher affinity for saturated acyl chains than for polyunsaturated ones [68]. Thus, in the presence of the hybrid 18:0–22:6PC, cholesterol can interact with both POPC and the hybrid omega-3 phospholipid, both bearing a saturated tail, conferring a significant order to the lipid bilayer.

As for the ordering effect at intermediate nitroxide-label positions in the presence of 18:0–22:6PC with respect to chol/POPC systems (Figure 3b), it could be related to the conformational freedom of the polyunsaturated chains. Indeed, DHA was proven to adopt multiple conformations, in particular bended conformations or two different extended stick-like conformations, i.e., the helical and the angle iron ones [69,70]. Previous findings indicated that cholesterol somehow stretches DHA [42]. Thus, the increased order may be related to the adoption of extended DHA conformations, which would allow a tighter packing [71] among them or with cholesterol than the bended POPC oleyl chain with its single *cis* double bond [34].

Conversely, cholesterol interactions with 22:6–22:6PC are weak, leaving the PUFA chains free to adopt disordered bent conformations, leading to an overall increased disorder of the lipid packing. This effect is relatively weak up to x_22:6–22:6PC_ = 0.6, while above this threshold a transition is observed; the lipid tails probably fold back, as highlighted by the dramatic drop of the S values (Figure 3a) and the bilayer thickness decreases. This transition occurs at a DHA content which can be reached only with the symmetric omega-3 phospholipid.

### 3.2. Cholesterol Suppresses Morphological Changes in 22:6–22:6PC-Rich Systems

Even above the transition to a less ordered bilayer observed at high omega-3 phospholipid content, deviations from the lamellar arrangement of lipids are ruled out by both SANS and NR results. SANS indicates that vesicular aggregates are present at all 22:6–22:6PC concentrations and that only very small rearrangements occur, specifically a slight decrease of lamellarity. Similarly, NR analysis confirms the presence of supported lipid bilayers at all the di-polyene concentrations; not only is no detachment observed, but bilayer features assure a very good support coverage in all cases, as indicated by area/lipid values. 

These results are in striking contrast to what was found when studying the effect of 22:6–22:6PC on the Ld bilayers formed by POPC [22]. In that case, above the threshold of x_22:6–22:6PC_ = 0.4, a massive structural reorganization was observed. Bending of polyunsaturated tails led to partial exposure of chain termini to the solvent and impaired formation of lamellar phases [22]. The presence of cholesterol appears to assure the persistence of a lamellar structure and suppress any morphological transition, even at very high 22:6–22–6PC concentrations. Such a result suggests that, even in the absence of a favourable interaction of cholesterol with the di-polyene 22:6–22:6PC (see the previous subsection), cholesterol is still molecularly dispersed among the phospholipids. Indeed, 22:6–22:6PC is not able to form lamellar phases in the presence of POPC when its molar fraction exceeds 0.4, and cholesterol also does not form lamellar phases. Thus, the formation of well-structured bilayers for chol/POPC/22:6–22:6PC x_22:6–22:6PC_ = 0.8 could be only due to the lipid mixtures considered as a whole, with these different lipids not separating one from the other.

### 3.3. Cholesterol Is Highly Soluble in 22:6–22:6PC-Rich Systems

The ability of 22:6–22:6PC-rich systems to form lamellar phases in the presence of cholesterol, in addition to the lack of cholesterol precipitation, seem to disagree with the very low cholesterol solubility reported in the literature for polyunsaturated systems. While in the case of the hybrid 18:0–22:6PC the maximum solubility of cholesterol has been reported to be around 55% [72], well above the concentration we used, for 22:6–22:6PC a low solubility, around 15%, has been reported by Brzustowicz et al. [72,73]. These low values have been the topic of scientific debate. Ibarguren et al. have indicated that it is possible to reach high cholesterol contents in bilayers of polyunsaturated di-arachidonic phosphatidylcholine (20:4–20:4PC) [74]. These discrepancies have been proposed to be related with the different preparation protocols used [75], and it has been hypothesized that at high cholesterol concentrations, meta-stable states form [76,77,78]. However, experimental investigations have clearly demonstrated that high cholesterol contents are stable with no precipitation observed up to over 10 weeks incubation at room temperature [75]. This stability is fully confirmed by our study, indicating molecular dispersion of cholesterol in all the investigated lipid mixtures and multi- and unilamellar vesicles (used for EPR and SANS experiments, respectively) as well as in supported bilayers (used for NR tests).

### 3.4. Domain Formation Does Not Occur in Lipid Mixtures Formed by Cholesterol and Polyunsaturated Phosphocholines

Cholesterol was not only believed to macroscopically separate, as discussed above, but also to form micro- or nanoscopic domains in the presence of polyunsaturated lipids. By means of NR, an increase of roughness was found in the case of chol/POPC/22:6–22:6PC x_22:6–22:6PC_ = 0.8. This behaviour has been previously suggested to be related to compositional and/or phase inhomogeneities [67]. However, the roughness value is not very different from those obtained for monocomponent homogeneous systems such as pure POPC bilayers [22,66]. Moreover, although NR and SANS do not directly probe domain formation within the plane of the bilayer, EPR with the spin probe approach has been used successfully to determine phase coexistence and domain formation [79,80,81], and our EPR results do not give indication of the domain formation due to cholesterol-polyunsaturated lipid demixing.

The lack of evidence for domain formation can be related to the investigated lipid systems; most studies in the literature have focused on phosphatidylethanolamine (PE) bearing polyunsaturated acyl chains [45] or on ternary mixtures containing not only polyunsaturated phospholipids, either PCs or PEs, and cholesterol, but also saturated lipids such as sphyngomyelin (SM) [54,82,83,84,85,86], a well-known component of lipid rafts [87]. Conversely, we focused on mixtures containing only PCs and cholesterol. Phase separation of lipids in model biomembranes and the consequent domain formation depend on both lipid headgroup and acyl chain properties. Distribution of cholesterol, with its very small hydrophilic headgroup and bulky hydrophobic portion, is strongly affected by the headgroup size of neighbouring lipids [88]. PC and PE headgroup structures differ since PC bears a trimethyl ammonium group hydrated with ~11.3 water molecules, while PE has an azaniumyl group and ~6.6 hydration water [89]. The relatively larger headgroup of PC including the hydration water results in a higher affinity for cholesterol compared to PE. This is due to the umbrella effect [34,90]. A phospholipid with a larger polar headgroup (and a smaller critical packing parameter, CPP) covers cholesterol more effectively, and thus prevents the hydrophobic core of the lipid bilayer from being exposed to water. PE has a smaller headgroup (CPP > 1) than PC (CPP = 1), and thus it has lower affinity towards cholesterol. Therefore, formation of a cholesterol-enriched domain seems to be a peculiarity of PE membranes rather than a general feature of phospholipid bilayers. Furthermore, PEs are also able to form inter-molecular hydrogen bonds between their headgroups (while PCs are not) further promoting formation of PE-rich domains with exclusion of cholesterol [88]. The PC preference of cholesterol was also proved by formation of cholesterol/PC-enriched microdomains in the polyunsaturated 18:0–22:6PC/18:0–22:6PE/18:0–22:6PS/cholesterol membranes [91].

As for the lipid mixtures also containing SM, the sphingolipid saturated acyl chains pack very well with the rigid cholesterol backbone. This bi-molecular building block is found to separate from polyunsaturated phospholipids. Such effect enhances the segregation of cholesterol into SM-rich/sterol-rich rafts, favouring DHA-rich domains via cholesterol exclusion. However, even in this case, it was found that DHA-containing PE behaves differently from DHA-containing PC with a minimal incorporation of the former and a substantial penetration of the latter into raft-like domains [75,92].

In this framework, our results conclusively indicate that no lipid segregation occurs in bilayers formed by cholesterol and polyunsaturated PC lipids.

### 3.5. Cholesterol Appears to Preserve a Canonical In-Leaflet Location in Polyunsaturated Systems

A debated issue concerning model membranes formed by both cholesterol and unsaturated lipids is the actual position of the cholesterol in the bilayer. Several papers by Wassall et al. [43,44,46,47] have suggested an in-plane position of cholesterol, lying between the two leaflets perpendicular to the bilayer axis. This initial assumption was corrected throughout several years; specifically, a tilted cholesterol position replaced the perfectly flat perpendicular position proposed previously. Such an unusual location was related to the low bilayer thickness rather than to the presence of unsaturated or polyunsaturated lipids. In this respect, the results we obtained point against a significant midplane location, also in the very thin chol/POPC/22:6–22:6PC with x_22:6–22:6PC_ > 0.8.

Particularly, CNO as a spin probe is expected to give straightforward indications on the cholesterol behaviour into omega-3 enriched bilayers and in no case did we observe a change of experienced polarity or mobility, as expected in the case of cholesterol relocating in the bilayer midplane, even at high omega-3 contents. Moreover, the poor interaction with lipid tails that results from an in-plane position is unlikely to assure the packing necessary to preserve a lamellar arrangement at high 22:6–22:6PC contents.

As a whole, these findings suggest that most of cholesterol preserves the canonical in-leaflet locations, likely held in place by the favourable intercalation among PC lipids, even at high omega-3 concentrations.

### 3.6. The Co-Presence of Cholesterol and Symmetric Polyunsaturated Phospholipids Induces the Formation of a New Disordered Lipid Organization within the Bilayer

**Figure 10:**
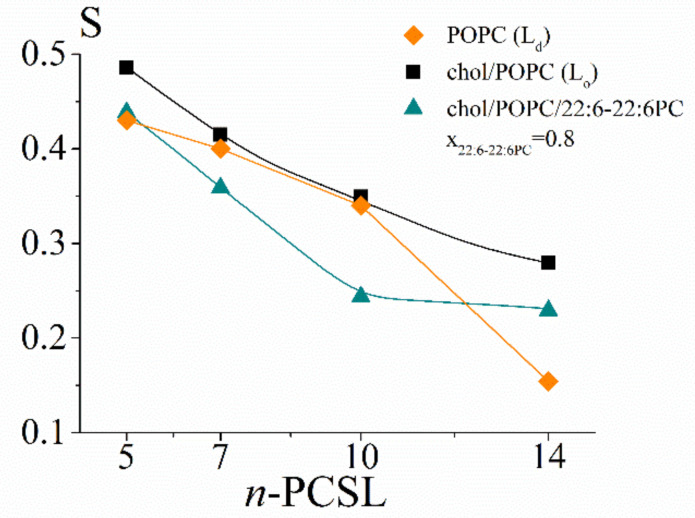
S profiles of POPC (orange diamonds), chol/POPC (black squares), and chol/POPC/22:6–22:6PC x_22:6–22:6PC_ = 0.8 (cyan triangles). Lines are guide to the eye. Since errors are within 1%, they may not be clearly visible in the figure.

Figure 10 illustrates the S profiles of POPC bilayers (which are in the Ld phase) and chol/POPC bilayers (which are in the Lo phase). It is evident that the latter bilayer is overall more ordered, in agreement with the known ordering effect of cholesterol. In particular, the main difference is observed to the terminus (probed by 14-PCSL), which is much more ordered in the Lo phase than in the Ld state, so that the ordering gradient is almost suppressed. 

Interestingly, by comparing the S profile of chol/POPC/22:6–22:6PC x_22:6–22:6PC_ = 0.8 with those of chol/POPC (Lo phase) and POPC (Ld phase) (Figure 10), it clearly emerges that the addition of very high concentrations of the di-polyene does not completely revert cholesterol effects and does not induce an Lo–Ld transition. Instead, a new lipid (dis)ordering is observed, with the intermediate segments of the tail being the most affected, while for the termini, an order intermediate between that of Ld and Lo phases is observed. Conversely, it is worth stressing that order in the outer region is the same of that of the canonical Ld phase, because this may explain the difficulty of detecting different liquid disordered phases; very often, lipid packing and order are investigated by using probes such as Laurdan [5] that are sensitive only to the properties of the bilayer outer region [93]. 

Our findings point against an oversimplified Ld/Lo description of the biological membrane; on the contrary, they fall into a new picture based on the existence of multiple states characterized by a wide range of lipid packings and molecular orders [6]. Such a picture better agrees with the incredible lipid diversity of biological membranes and to the fine control that they can exert on a variety of biological processes [5]. 

## 4. Materials and Methods

### 4.1. Materials

Dichloromethane and methanol (HPLC-grade purity) were purchased from Merck (Darmstadt, Germany); cholesterol (chol), D_2_O (99% purity) and Phosphate Buffer Saline (PBS) tabs were purchased from Sigma Aldrich (St. Louis, MO, USA). 1-Palmitoyl-2-oleoyl-*sn*-glycero-3-phosphatidylcholine (POPC), 1-d31-Palmitoyl-2-oleoyl-sn-glycero-3-phosphatidylcholine (d31-POPC), 1-stearoyl-2-docosahexaenoyl-sn-glycero-3-phosphocholine (18:0–22:6PC), 1,2-didocosahexaenoyl-*sn*-glycero-3-phosphocholine (22:6–22:6PC), spin-labelled phosphatidylcholines (1-palmitoyl-2- stearoyl-(*n*-doxyl)-*sn*-glycero-3-phosphocholine, *n*-PCSL) with the nitroxide group in the positions 5, 7, 10 and 14 of the acyl chain, and 25-doxyl-cholesterol (CNO) were purchased from Avanti Polar Lipids (Birmingham, AL, USA). Molecular structures of the lipids used in this study are displayed in Figures S1 and S2 (Supporting Material).

### 4.2. Sample Preparation

All the samples were prepared by mixing appropriate amounts of lipids, dissolved in a dichloromethane–methanol mixture (2:1 *v*/*v*, 10 mg/mL lipid concentration) in a round-bottom test tube. In the samples prepared for neutron reflectivity experiments, POPC was replaced with deuterated d31-POPC. For EPR measurements, spin-probes (i.e., either *n*-PCSL or CNO) were added to the lipid mixture (1% by mole on the total lipids) by mixing appropriate amounts of a spin-probe solution in ethanol with the lipid organic mixture. A thin lipid film was formed by evaporating the solvents with dry nitrogen gas, and final traces of solvents were removed by subjecting the sample to vacuum desiccation for at least 3 h. The samples were finally hydrated with PBS solution in water, pH = 7.4, thus obtaining 1 mM multi lamellar vesicle (MLV) suspensions, repeatedly vortexed, and slightly sonicated. These samples were directly used for EPR measurements. For SANS experiments, PBS prepared with heavy water was used as a solvent, and large unilamellar vesicles (LUVs) were obtained by repeatedly extruding the MLV suspensions through a 100 nm pore size polycarbonate membrane. All the samples were monitored for at least 24 h, with no change being detected. Omega-3 breakdown initiated by free-radical formation and subsequent propagation by chain-reaction was monitored as described elsewhere [22]. Neutron reflectivity measurements were performed on lipid bilayers deposited on monocrystalline silicon supports through the vesicle fusion protocol. For this purpose, a 0.5 mM vesicle suspension prepared as described above was injected in the solid-liquid reflectometry flow cell and equilibrated with the silicon support surface for 30 min. Then pure D_2_O was injected into the cell, leading to the rupture of the vesicles and formation of the desired planar lipid bilayers.

All experiments were performed at room temperature (about 25 °C), which is well above the transition temperature of POPC (−3.7 °C) [94,95]. Also considering the cholesterol induced increase of the transition temperature, in these conditions all the systems are in a fluid, liquid ordered phase [56].

### 4.3. Electron Paramagnetic Resonance (EPR) Spectroscopy

EPR spectra were recorded with a 9 GHz Bruker Elexys E-500 spectrometer (Bruker, Rheinstetten, Germany). The samples (20 μL) were inserted in glass capillaries with an internal diameter of 1.2 mm, which were flame-sealed. Then, capillaries containing MLV suspensions were placed in a standard 4 mm quartz sample tube containing light silicone oil for thermal stability and analysed with no further treatment. All the measurements were performed at 25 °C. Spectra were recorded using the following instrumental settings: sweep width, 100 G; resolution, 1024 points; time constant, 20.48 ms; modulation frequency, 100 kHz; modulation amplitude, 1.0 G; incident power, 6.37 mW. Several scans, at least eight, were accumulated to improve the signal-to-noise ratio.

### 4.4. Analysis of EPR Spectra

The EPR spectra of the *n*-PCSL and CNO probes in liposomes constituted by cholesterol, POPC, and either 18:0–22:6PC or 22:6–22:6PC at different relative percentages (see Table 1 and the spectra in Figures S3–S5, Supporting Material) were simulated using the well-known program of Budil and Freed [96]. The approach used for the computation of the EPR spectra was used previously by some of us [58,59]; in particular, we employed it for similar systems composed of POPC and 22:6–22:6PC [22], so we chose it for sake of comparison. The parameters agree with those obtained in these previous studies. The computation strategy considers the high number of parameters needed to compute the EPR spectra of such complex systems. Changing all parameters strongly decreases the accuracy in the evaluation of each parameter, and the impossibility of getting reliable structural and dynamical information in a comparative manner. The goal of this study is to follow the change of each of few informative parameters by changing the molar ratios in the chol/POPC/22:6–22:6PC and chol/POPC/18:0–22:6PC systems. The reliability of the method resides in the very good fitting between the experimental and the computed line shapes, as indicated in Figures S6–S8, by fixing all parameters except for the selected/informative ones, as described in the following:

- the g_ii_ values for the coupling between the electron spin and the magnetic field. Those giving the best fitting for all spectra were: g_ii_ = 2.0075, 2006, 2.003 in the case of n-PCSL spectra, and g_ii_ = 2.014, 2.006, 2.0015 in the case of CNO spectra;
- the A_ii_ values for the coupling between the electron spin and the nitrogen nuclear spin. They were also maintained constant for each spin probe, since the same values well fitted all the spectra: A_ii_ = 7.1 G, 7.1 G, 29.7 G, <A> = (Axx + Ayy + Azz)/3 = 14.7 G, in the case of 5-PCSL spectra; A_ii_ = 7.1 G, 7.1 G, 29.0 G, <A> = 14.4 G, in the case of 7-PCSL spectra; A_ii_ = 7.1 G, 7.1 G, 28.5 G, <A> = 14.2 G, in the case of 10-PCSL spectra; A_ii_ = 7.1, 7.1, 28.0 G, <A> = 14.1 G, in the case of 14-PCSL spectra; A_ii_ = 5.0, 5.0, 33.0 G, <A> = 14.3 G, in the case of CNO spectra;
- the correlation time for the rotational diffusion motion of the probes, τ. To improve the fitting between the experimental and the computed line shapes, it was necessary to include in the calculation an anisotropy of motion (different parallel and perpendicular τ values, namely τ_par_ and τ_perp_), also considering a tilt of the rotational axis. However, in the case of all the spin probes, the fitting was good by taking constant both the τ_par_ value (13.2 ns, indicative of the steric hindrance of the chain in its parallel direction with respect to the p-orbital hosting the unpaired electron) and the tilt angle (70°). Therefore, the main parameter τ changing from one to another system is τ_perp_;
- the order parameter, indicated as S, which measures the orientational ordering of the labelled segment of the acyl chain with respect to the normal to the bilayer surface. This parameter changes from one system to another.

Several other computational approaches were tried to analyse the EPR spectra in a comparative manner and this one was the most informative and reliable. The parameters, both the fixed ones and the variable ones, were selected considering the physical meaning of them, based on the structures and organization of the compounds. 

The error in the parameters derives from the fitting between the experimental and the computed line shapes: values exceeding the error produced a worse fitting between the experimental and the computed spectra.

### 4.5. Small Angle Neutron Scattering (SANS)

SANS measurements were performed at 25 °C with the KWS-1 diffractometer operated by the Jülich Centre for Neutron Science (JCNS) at the FRMII source located at the Heinz Maier-Leibnitz Zentrum (MLZ), Garching (Germany) [97,98]. For all the samples, neutrons with a wavelength (λ) of 5 Å and Δλ/λ ≤ 0.1 were used. A two-dimensional array detector at three different wavelength (λ)/collimation (C)/sample-to-detector (D) distance combinations (λ 5 Å/C 8 m/D 2 m, λ 5 Å/C 8 m/D 8 m, and λ 5 Å/C 20 m/D 20 m) were selected. The scattering intensity was collected in the range of the modulus of the scattering vector, Q = 4πsin(θ/2)/λ, between 0.0023 Å^−1^ and 0.45 Å^−1^. Here λ and θ represent the wavelength of the neutron beam and scattering angle, respectively. Each sample and solvent background was placed in capped 2 mm path length quartz cuvettes to prevent solvent evaporation and exchange with atmospheric water vapour.

### 4.6. Analysis of SANS Data

The raw SANS data were corrected for background and empty cell scattering. Detector efficiency correction, radial average, and transformation to absolute scattering cross sections dΣ/dΩ were made with a secondary plexiglass standard. The absolute scattering cross section data dΣ/dΩ were plotted as function of Q. The dependence of dΣ/dΩ from the scattering vector can be summarized as:

$$\frac{d\Sigma }{d\Omega }=n_{p}P\left(Q\right)S\left(Q\right)+{\left(\frac{d\Sigma }{d\Omega }\right)}_{\mathrm{incoh}}$$

where n_p_ is the number of scattering objects, P(Q) and S(Q) are respectively the form factor and the structure factor. The last term considers the incoherent scattering mostly due to the presence of hydrogen atoms within the sample. Structural information can be extrapolated by choosing an appropriate model to fit the experimental data. 

A detailed quantitative analysis was performed by fitting the data using the SASView program [62]. In all the cases, no structure factor was considered. For the form factor, several models, including a vesicular one, were tested. A lamellar stack paracrystal model [63], usually employed for simulation of large multilamellar vesicles, satisfactorily fit the data relative to the lipid mixtures chol/POPC and chol/POPC/22:6–22:6PC with x_22:6–22:6PC_ = 0.2 and 0.8. Equations are reported in the Supporting Material.

### 4.7. Neutron Reflectivity (NR)

Neutron reflectivity measurements were performed on Maria reflectometer of the Jülich Centre for Neutron Science at MLZ (Garching, Germany) [99], varying the incidence angle of the incoming beam and using two different wavelengths: 12 Å for the low-Q region and 6 Å for the high-Q region up to 0.20 Å^−1^, with a wavelength spread of Δλ/λ = 0.1.

The specular reflection at the silicon/water interface, R, defined as the ratio between the reflected intensity and the transmitted intensity through the incoming medium of a neutron beam, is measured as a function of the surface normal component, Q_z_, of the wave vector transfer, Q. R(Q_z_) is related to the scattering length density (SLD) across the interface, $\rho \left(z\right)$, which depends on the composition of the adsorbed species. The neutron scattering length density, $\rho \left(z\right)$, is defined by the following relation:

$$\rho \left(z\right)=\underset{j}{\sum }n_{j}\left(z\right)b_{j}$$

where $n_{j}\left(z\right)$ is the number of nuclei per unit volume and $b_{j}$ is the scattering length of nucleus j. The scattering lengths of the constituent fragments of any species adsorbed at the surface are the fundamental quantities from which the interfacial properties and microstructural information on the lipid bilayer are derived. Measurements of the same system in different solvent contrasts greatly enhance the sensitivity of the technique [100], so all the samples were measured using D_2_O, H_2_O, and SiMW (silicon-matched water) as solvent contrasts. SiMW (ρ = 2.07 × 10^−6^ Å^−2^) is a mixture of 38 vol % D_2_O (ρ = 6.35 × 10^−6^ Å^−2^) and 62 vol % H_2_O (ρ = −0.56 × 10^−6^ Å^−2^) with the same refraction index for neutrons as a bulk silicon.

### 4.8. Analysis of NR Data

Neutron reflectivity curves were analysed with a fitting procedure based on parameterized volume fraction profiles of all chemical components (silicon, silicon oxide, water, lipid tails, and headgroups) as detailed in a previous study [22]. The validity of the method was also proven in previous studies [64,65,101].

The lipid bilayer was modelled as three slabs: two identical slabs corresponding to the two head regions sandwiching one single slab corresponding to the tail region. The headgroup and tail physical parameters (i.e., molecular volume and *SLD*) are calculated by mixing values of single components reported in Table S2 (Supporting Material) using suitable mol/mol ratios.

This analysis allowed us to obtain, beyond structural parameters of the bilayer, such as tail and headgroup thickness, the surface excess of lipids adsorbed onto the W/Si interface, which represents the lipids adsorbed per unit area, and the global roughness of the bilayer, obtained by convolving distribution profiles of headgroups and tails by a Gaussian function as detailed previously [22]. Analysis of NR data and error estimation is briefly detailed in the Supplementary Material.

## 5. Conclusions

Through an extensive characterization of lipid mixtures containing cholesterol, POPC, and variable contents of polyunsaturated phosphocholines, we indicate that several assumptions on the behaviour of systems composed of cholesterol and symmetric di-polyenes, although strongly rooted in the literature, need reconsideration. Our results indicate that cholesterol has a significant solubility in mixture with polyunsaturated phosphocholines and does not phase-separate in distinct domains. Moreover, it mostly preserves its canonical position intercalated with lipid tails rather than relocate in the mid-plane between leaflets. These features do not vary much depending on the nature of the omega-3 lipid, i.e., symmetric dipolyene or hybrid monopolyene.

However, symmetric and hybrid polyunsaturated lipids differently affect some microstructural and dynamic features of lipid bilayers formed by POPC and cholesterol. While the hybrid PUFA lipid induces a weak ordering of the lipid tails even at high PUFA concentrations, the symmetric lipid disorders them in a dose dependent manner. Particularly, a DHA threshold exists, above which a dramatic tail rearrangement occurs, connected to a much higher rotational mobility of the tail termini and to their possible folding. Interestingly, this threshold can be reached only with the symmetric omega-3 phospholipid supporting the hypothesis that symmetric omega-3 phospholipids, which are present in much lower proportions in most tissues, but are particularly abundant in specific cell types, play very specific roles.

Overall, our findings suggest that the ratio between hybrid polyunsaturated lipids, which are widely distributed in natural membranes, and the rare symmetric ones could exert a fine control of membrane-related phenomena through local, rather than global, changes of the membrane properties and this may represent an advantage in terms of rapidity and energy costs [102]. 

We also prove that addition of symmetric polyunsaturated lipids to Lo systems effectively reverses some cholesterol-induced effects, including a thinning and disordering of the bilayer. Conversely, the presence of cholesterol allows the lipid aggregates to keep a bilayer structure, even at a concentration at which PUFA lipids would promote a phase transition to non-lamellar phases [22].

The delicate balance between PUFA and cholesterol effects leads to the stabilization of lipid bilayers whose features are ascribable neither to a Lo nor to a canonical Ld state and must rather be considered as signature of a different lipid bilayer organization, characterized by a disorder mainly localized in the intermediate chain length region. This should prompt us to start thinking beyond the simple Lo/Ld distinction and embrace the idea of the biomembrane as a mosaic of many different more or less ordered phases. 

## Figures and Tables

**Table 1:** Lipid compositions of the chol/POPC/18:0–22:6PC and chol/POPC/22:6–22:6PC lipid mixtures investigated in the present work.

	Molar Fraction of Polyunsaturated Lipids with Respect to Total Phosphocholines	Molar Fraction with Respect to Total Lipids				Molar Fraction of DHA with Respect to Total Phosphocholine Tails
	$x_{22:6–22:6PC}=\frac{mol_{22:6–22:6PC}}{mol_{PC}}$ $x_{18:0–22:6PC}=\frac{mol_{18:0–22:6PC}}{mol_{PC}}$	**Cholesterol**	**POPC**	**22:6–22:6PC**	**18:0–22:6PC**	$x_{DHA}=\frac{mol_{DHA}}{mol_{PC\;tails}}$
**chol/POPC**	x_22:6–22:6PC_ = 0x_18:0–22:6PC_ = 0	0.4	0.6	0	0	x_DHA_ = 0
**chol/POPC/** **22:6–22:6PC**	x_22:6–22:6PC_ = 0.2	0.4	0.48	0.12	0	x_DHA_ = 0.2
	x_22:6–22:6PC_ = 0.4	0.4	0.36	0.24	0	x_DHA_ = 0.4
	x_22:6–22:6PC_ = 0.5	0.4	0.3	0.3	0	x_DHA_ = 0.5
	x_22:6–22:6PC_ = 0.6	0.4	0.24	0.36	0	x_DHA_ = 0.6
	x_22:6–22:6PC_ = 0.8	0.4	0.12	0.48	0	x_DHA_ = 0.8
**chol/22:6–22:6PC**	x_22:6–22:6PC_ = 1	0.4	0	0.6	0	x_DHA_ = 1
**chol/POPC/** **18:0–22:6PC**	x_18:0–22:6PC_ = 0.2	0.4	0.48	0	0.12	x_DHA_ = 0.1
	x_18:0–22:6PC_ = 0.4	0.4	0.36	0	0.24	x_DHA_ = 0.2
	x_18:0–22:6PC_ = 0.5	0.4	0.3	0	0.3	x_DHA_ = 0.25
	x_18:0–22:6PC_ = 0.6	0.4	0.24	0	0.36	x_DHA_ = 0.3
	x_18:0–22:6PC_ = 0.8	0.4	0.12	0	0.48	x_DHA_ = 0.4
**chol/18:0–22:6PC**	x_18:0–22:6PC_ = 1	0.4	0	0	0.6	x_DHA_ = 0.5

**Table 2:** Structural parameters as derived from model fitting of SANS profiles. Errors as derived from fitting are reported.

	Thickness (Å)	N Layers	<D> (Å)	σD/<D>	Polydispersity on Thickness
**chol/POPC**	38.09 ± 0.05	4.424 ± 0.005	105.14 ± 0.06	1.311 ± 0.002	0.05
**chol/POPC/22:6–22:6PC** **x_22:6–22:6PC_ = 0.2**	38.67 ± 0.06	3.801 ± 0.003	94.73 ± 0.06	1.801 ± 0.002	0.1
**chol/POPC/22:6–22:6PC** **x_22:6–22:6PC_ = 0.8**	34.60 ± 0.09	2.698 ± 0.006	110.1 ± 0.2	1.877 ± 0.008	0.05

**Table 3:** Lipid bilayer structural parameters corresponding to the best fit of Neutron Reflectivity profiles. In brackets the confidence interval of 95% is reported.

	Headrgroup Thickness (Å)	Tail Thickness (Å)	Bilayer Roughness (Å)	Area/Lipid (Å^2^)
**chol/POPC**	7.2 (6.8–8.1)	35.8 (33.6–37.8)	2.9 (2.4–5.6)	45 ± 1
**chol/POPC/22:6–22:6PC** **x_22:6–22:6PC_ = 0.2**	8.5 (7.7–9.3)	27.2 (25.9–30.5)	0.7 (0.6–3.0)	84 ± 3
**chol/POPC/22:6–22:6PC** **x_22:6–22:6PC_ = 0.8**	13.0 (13.0–13.0)	23.3 (22.7–25.5)	8.7 (7.2–9.2)	60 ± 4
**chol/POPC/18:0–22:6PC** **x_22:6–22:6PC_ = 0.4**	12.4 (11.3–12.5)	30.6 (29.1–32.4)	3.4 (2.8–4.5)	77 ± 3
**18:0–22:6PC/chol**	7.8 (7.1–8.7)	29.1 (28.5–29.8)	3.0 (2.5–3.6)	61 ± 1

## Data Availability

Not applicable.

## Supplementary Materials

The following supporting information can be downloaded at: https://www.mdpi.com/article/10.3390/ijms23105322/s1.
