# Supplementary material for: Order vs. Disorder: Cholesterol and Omega-3 Phospholipids Determine Biomembrane Organization
Source: Int J Mol Sci. 2022 May 10;23(10):5322. doi: 10.3390/ijms23105322 (PMC9140907; doi:10.3390/ijms23105322)
Supplement: Supplementary file 1 [file ijms-23-05322-s001.zip › ijms-1693708-supplementary.pdf]

Supplementary Material

To

Order *vs*. disorder: cholesterol and omega-3 phospholipids determine biomembrane organization

Augusta de Santis,[1,2] Ernesto Scoppola,[3] Maria Francesca Ottaviani,[4] Alexandros Koutsioubas,[5] Lester C Barnsley,[5,6] Luigi Paduano, [1,2] Gerardino D'Errico, [1,2,*] Irene Russo Krauss[1,2,*]

[1] *Department of Chemical Science, University of Naples Federico II, Naples, Italy*

[2] *CSGI (Consorzio per lo Sviluppo dei Sistemi a Grande Interfase), Florence, Italy*

[3] *Max Planck Institut für Kolloid und Grenzflächenforschung, Potsdam, Germany*

[4] *Department of Pure and Applied Sciences, University of Urbino, Urbino, Italy*

[5] *Jülich Centre for Neutron Science (JCNS) at Heinz Maier-Leibnitz Zentrum (MLZ), Garching, Germany*

[6] *Present address: Australian Synchrotron, ANSTO, Clayton 3168, Australia*

* Correspondence: G.D.E. gerardino.derrico@unina.it; I.R.K. irene.russokrauss@unina.it

Analysis of SANS data

In the case of the lamellar stack paracrystal model [1], the scattering intensity *I(q)* was calculated as

$$I(q) = 2\pi\Delta\rho^2\Gamma_m \frac{P_{bil}(q)}{q^2} Z_N(q)$$

where $\Delta\rho$ is the contrast, Γ_m is the volume fraction of the material in the bilayer and P_{bil} is the form factor of the bilayer, approximated as the cross section of an infinite, planar bilayer of thickness according to

$$P_{bil}(q) = \left(\frac{sin\left(^{qt}/_{2}\right)}{^{qt}/_{2}}\right)^2$$

Finally, Z_N(q) describes the interference effects for aggregates consisting of more than one bilayer, and depends on the number of bilayers N, the average distance between adjacent layers $\langle D \rangle$ and the relative standard deviation of the Gaussian layer distance distribution $\sigma D/\langle D \rangle$.

Analysis of NR data and error estimation

NR data were fitted by using an in-house Python software based on Metropolis Monte-Carlo with a simulated annealing algorithm.

For a given set of parameters $\Lambda^{(i)} = (\lambda_1^{(i)}, \dots, \lambda_N^{(i)})$ a penalty function $\chi^2_{(i)}$ as

$$\chi^2(\Lambda^{(i)}) = \chi^2_{(i)} = \sum_{m=1}^{M} \left(\frac{\mathrm{R}_e(q_{z,m}) - \mathrm{R}_t(q_{z,m}, \Lambda^{(i)}))}{\Delta\mathrm{R}_e(q_{z,m})}\right)^2$$

representing the square distance between model and data. $\mathrm{R}_e(q_z)$, $\Delta\mathrm{R}_e(q_z)$, $\mathrm{R}_t(q_z, \Lambda^{(i)})$ represent the experimental data, the corresponding uncertainty and the model data, respectively. In general, the parameter set minimizing the penalty function is selected as best-fit model $\Lambda^{(best)}$.

In the Metropolis Monte-Carlo algorithm a link between penalty function and associated probability is defined as follows

$$P(\Lambda^{(i)}) = exp\left[-\frac{\chi^2_{(i)}}{E}\right]$$

and for a given E value, the larger χ^2 the smaller the probability. If the model $\Lambda^{(i+1)}$ is obtained by a perturbation of one of the parameters $\Lambda^{(i)}$, the associated probability of acceptance or rejection can be calculated by the mean of the *detailed balance* such that:

$$P_{i,i+1} = P\left(\Lambda^{(i)} \rightarrow \Lambda^{(i+1)}\right) = \frac{P\left(\Lambda^{(i+1)}\right)}{P(\Lambda^{(i)})} = exp\left[-\frac{\left(\chi^2_{(i+1)} - \chi^2_{(i)}\right)}{E}\right]$$

It is obvious that independently of E, if $\chi^2_{(i+1)} \leq \chi^2_{(i)} \implies P_{i,i+1} \geq 1$. On the other hand, during a fitting procedure based on the Metropolis Monte-Carlo, the choice of E is relevant for accepting or rejecting the parameter set $\Lambda^{(i+1)}$. For this reason, in the particular case of the work presented here, E is varied with a simulated annealing approach during the fitting procedure.

Additionally, the phase space (i.e. χ^2 landscape) is defined by the parameter set, data and related uncertainties. By assuming that the minimum $\chi^2_{(best)}$ represents the minimum in the phase space corresponding by the best-match parameter set $\Lambda^{(best)}$, we can define the normalized probability of each $\Lambda^{(i)}$ tested during the fitting procedure as

$$\tilde{P}\left(\Lambda^{(i)}\right) = \frac{P\left(\Lambda^{(i)}\right)}{P(\Lambda^{(best)})} = exp\left[-\frac{\chi^2_{(i)}}{\chi^2_{(best)}}\right] \cdot exp\left[\frac{\chi^2_{(best)}}{\chi^2_{(best)}}\right] = exp\left[-\frac{\left(\chi^2_{(i)} - \chi^2_{(best)}\right)}{\chi^2_{(best)}}\right]$$

The latter equation becomes a powerful tool for the estimation of parameters uncertainties allowing the definition of the probability associated to a given parameter set tested during the fitting procedure. Moreover, in combination with the Metropolis Monte-Carlo and Simulated annealing algorithms with a random exploration of parameters, it is possible to define probability maps for those observables resulting from the combination of several parameters. This approach represents a self-consistent method for parameter uncertainties estimation based on the Metropolis Monte-Carlo definition of probabilities.

Table S1 Hyperfine coupling constant *A*, order parameter *S* and correlation time *τ*, for 5-PCSL, 14-PCSL and CNO as derived from EPR spectra simulation. Errors are within 1%.

		5-PCSL			14-PCSL			CNO		
		**A (G)**	**S**	**τ (ns)**	**A (G)**	**S**	**τ (ns)**	**A (G)**	**S**	**τ (ns)**
**chol/POPC**	$x_{22:6\text{-}22:6PC} = 0$ $x_{18:0\text{-}22:6PC} = 0$	14.67	0.48598	11.0	14.10	0.27944	3.0	14.33	0.23	0.33
**chol/POPC/ 22:6-22:6PC**	$x_{22:6\text{-}22:6PC} = 0.2$	14.67	0.4785	10.8	14.10	0.26729	2.8	14.33	0.227	0.33
	$x_{22:6\text{-}22:6PC} = 0.4$	14.67	0.4785	10.8	14.10	0.26449	2.5	14.33	0.218	0.33
	$x_{22:6\text{-}22:6PC} = 0.5$	14.67	0.4757	10.73	14.10	0.26075	2.3	14.33	0.21	0.33
	$x_{22:6\text{-}22:6PC} = 0.6$	14.67	0.47009	10.6	14.10	0.25607	2.1	14.33	0.205	0.33
	$x_{22:6\text{-}22:6PC} = 0.8$	14.67	0.43832	10.3	14.10	0.22897	1.0	14.33	0.195	0.33
**chol/22:6-22:6PC**	$x_{22:6\text{-}22:6PC} = 1$	14.67	0.40561	10.0	14.10	0.2	0.08	14.33	0.17	0.33
**chol/POPC/ 18:0-22:6PC**	$x_{18:0\text{-}22:6PC} = 0.2$	14.67	0.48411	10.8	14.10	0.27904	3.0	14.33	0.23052	0.33
	$x_{18:0\text{-}22:6PC} = 0.4$	14.67	0.48318	10.7	14.10	0.2785	2.95	14.33	0.228	0.33
	$x_{18:0\text{-}22:6PC} = 0.5$	14.67	0.48318	10.7	14.10	0.2785	2.9	14.33	0.2274	0.33
	$x_{18:0\text{-}22:6PC} = 0.6$	14.67	0.48318	10.7	14.10	0.2785	2.9	14.33	0.22745	0.33
	$x_{18:0\text{-}22:6PC} = 0.8$	14.67	0.48318	10.7	14.10	0.27664	2.9	14.33	0.22614	0.33
**chol/18:0-22:6PC**	$x_{18:0\text{-}22:6PC} = 1$	14.67	0.48411	10.8	14.10	0.26636	2.85	14.33	0.22338	0.33

Table S2 Scattering Length Densities (SLDs) and Molecular Volumes (V) used as input values for the Neutron Reflectivity fitting procedure.

Molecule	Chemical Formula	SLD [10^{-6} Å^{-2}]	V [Å^3]
**Heavy Water**	D_2O	6.3752	30.0428
**Light Water**	H_2O	-0.5594	29.9757
**POPC (Tail)**	$C_{32}H_{64}$	-0.2899	919.614
**POPC (Headgroup)**	$C_{10}H_{18}PO_8N$	1.8652	322.100
**Cholesterol (Tail)**			
**Cholesterol (Headgroup)**			
**22:6-22:6PC (Tail)**	$C_{42}H_{62}$	0.6543	722.940
**22:6-22:6PC (Headgroup)**	$C_{10}H_{18}PO_8N$	1.8652	322.100
**18:0-22:6PC (Tail)**			
**18:0-22:6PC (Headgroup)**	$C_{10}H_{18}PO_8N$	1.8652	322.100
**Silicon**	Si	2.0754	20.000
**Silicon Oxide**	SiO_2	3.2698	48.200

POPC

Cholesterol

18:0-22:6PC

22:6-22:6PC

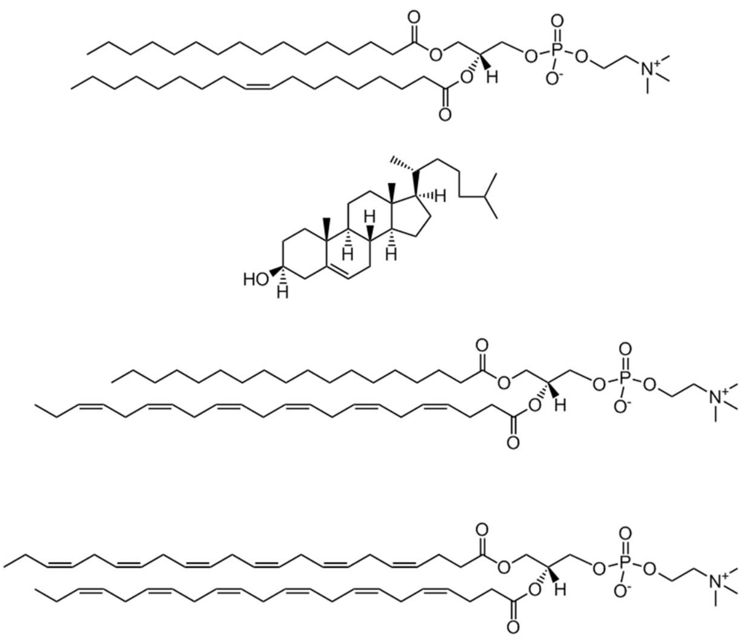

Figure S1 Chemical structure of the natural lipids employed in this study: 1-palmitoyl-2-oleoyl-sn-glycero-3-phosphocholine (POPC), cholesterol, 1-stearoyl-2-docosahexaenoyl-sn-glycero-3-phosphocholine (18:0-22:6PC), 1,2-didocosahexaenoyl-sn-glycero-3-phosphocholine (22:6-22:6PC)

d31-POPC

5-PCSL

14-PCSL

CNO

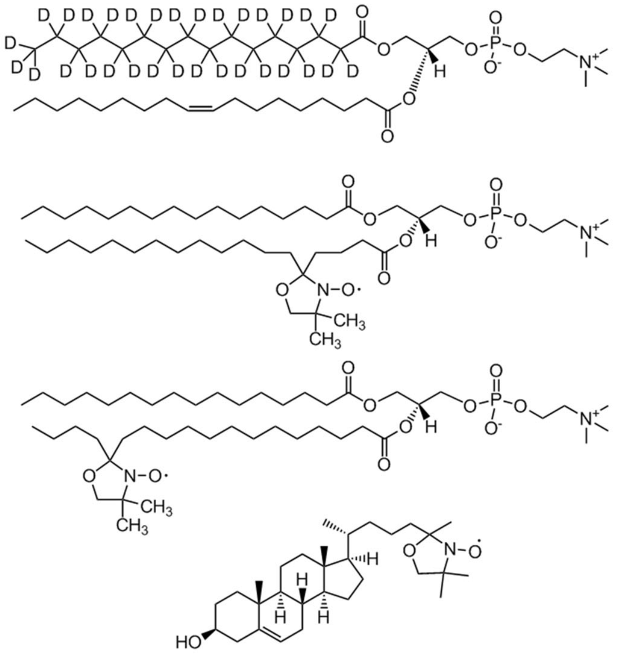

Figure S2 Chemical structure of the lipid probes employed in this study: 1-palmitoyl-d31-2-oleoyl-sn-glycero-3-phosphocholine (d31-POPC), 1-palmitoyl-2-stearoyl-(5-doxyl)-sn-glycero-3-phosphocholine (5-PCSL), 1-palmitoyl-2-stearoyl-(14-doxyl)-sn-glycero-3-phosphocholine (14-PCSL), 25-doxyl-cholesterol (CNO)

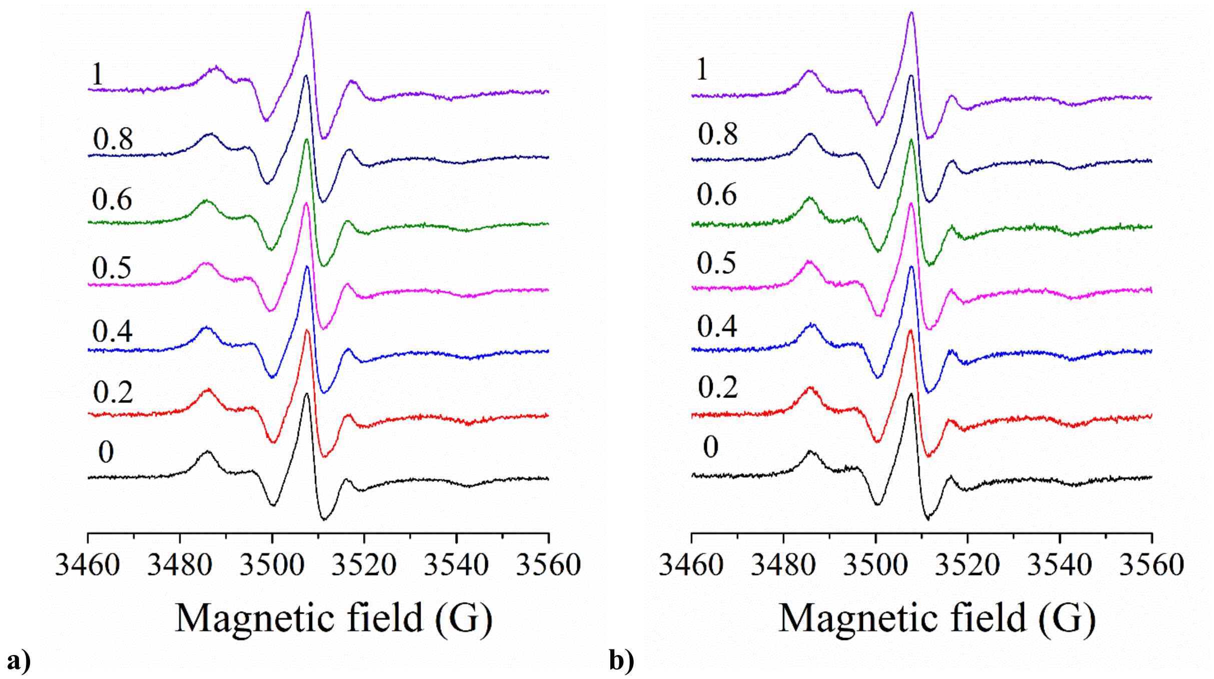

Figure S3 EPR spectra of 5-PCSL in (**a**) chol/POPC/22:6-22:6PC and (**b**) chol/POPC/18:0-22:6PC systems with $x_{22:6\text{-}22:6PC}$ and $x_{18:0\text{-}22:6PC}$ ranging from 0 to 1.

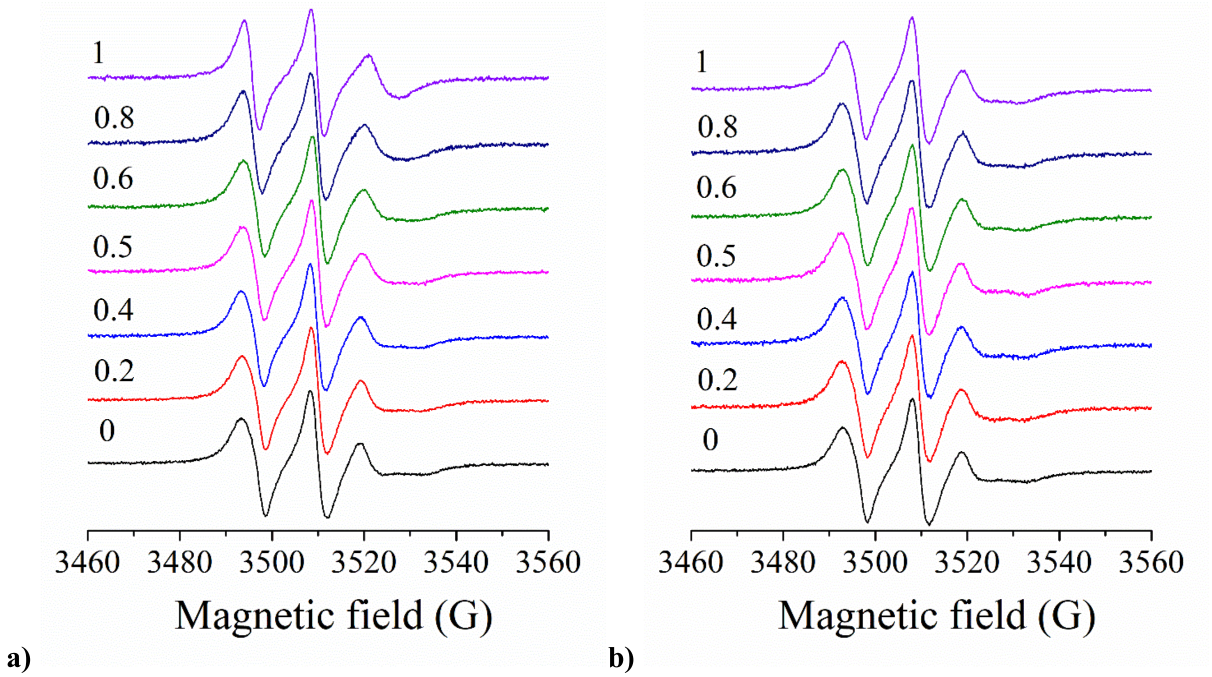

Figure S4 EPR spectra of 14-PCSL in (**a**) chol/POPC/22:6-22:6PC and (**b**) chol/POPC/18:0-22:6PC systems with $x_{22:6\text{-}22:6PC}$ and $x_{18:0\text{-}22:6PC}$ ranging from 0 to 1.

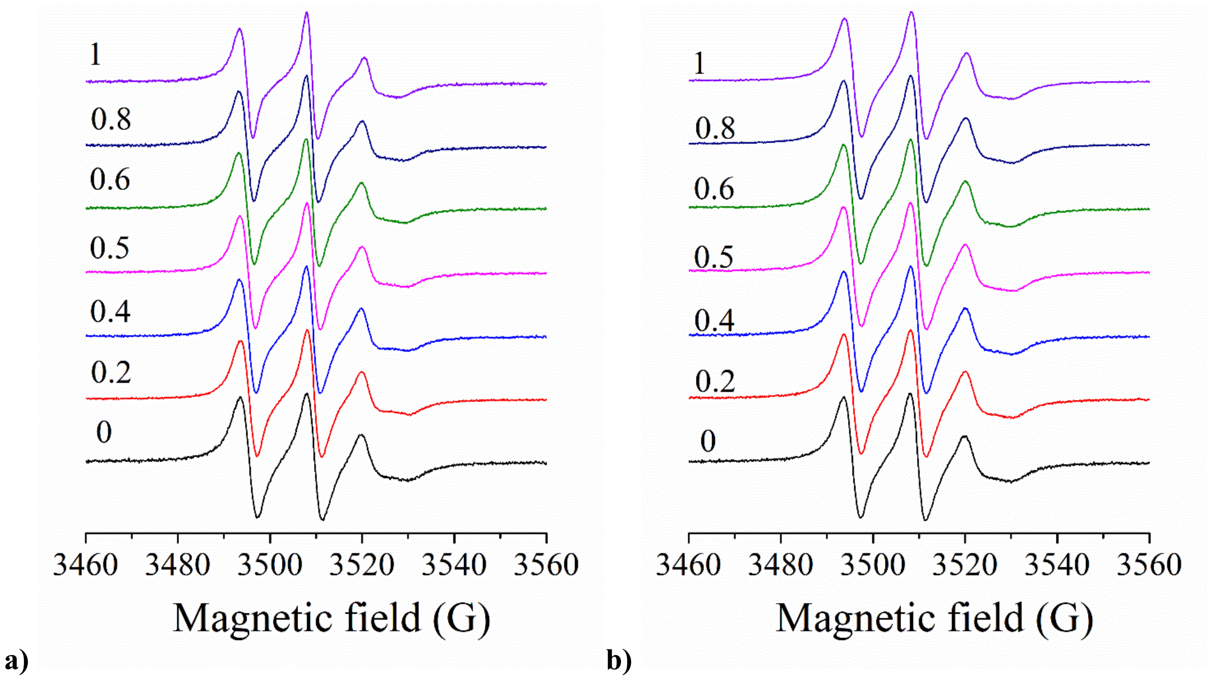

Figure S5 EPR spectra of CNO in (**a**) chol/POPC/22:6-22:6PC and (**b**) chol/POPC/18:0-22:6PC systems with $x_{22:6\text{-}22:6PC}$ and $x_{18:0\text{-}22:6PC}$ ranging from 0 to 1.

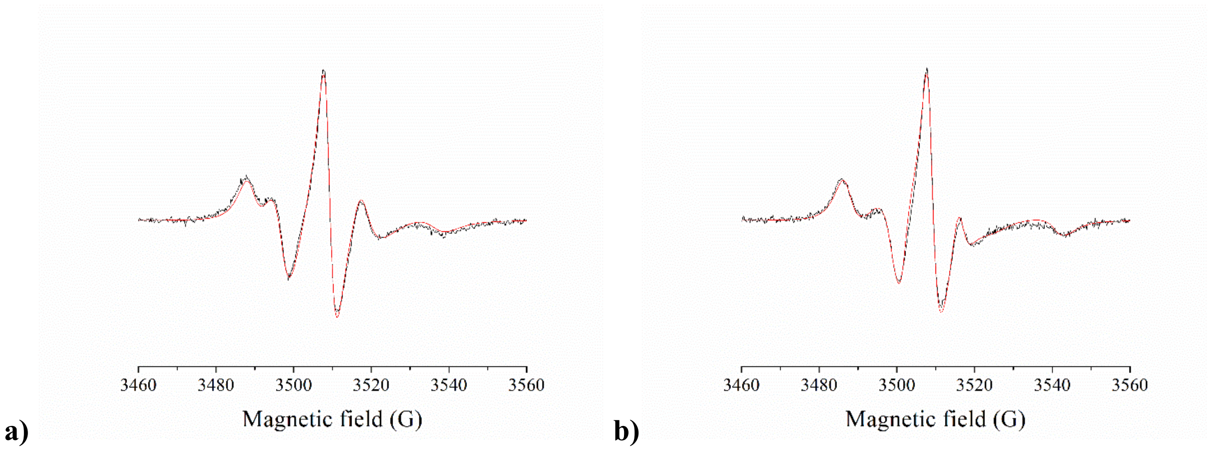

Figure S6 Comparison between experimental (black lines) and computed (red lines) EPR spectra of 5-PCSL in (**a**) chol/22:6-22:6PC and (**b**) chol/18:0-22:6PC systems.

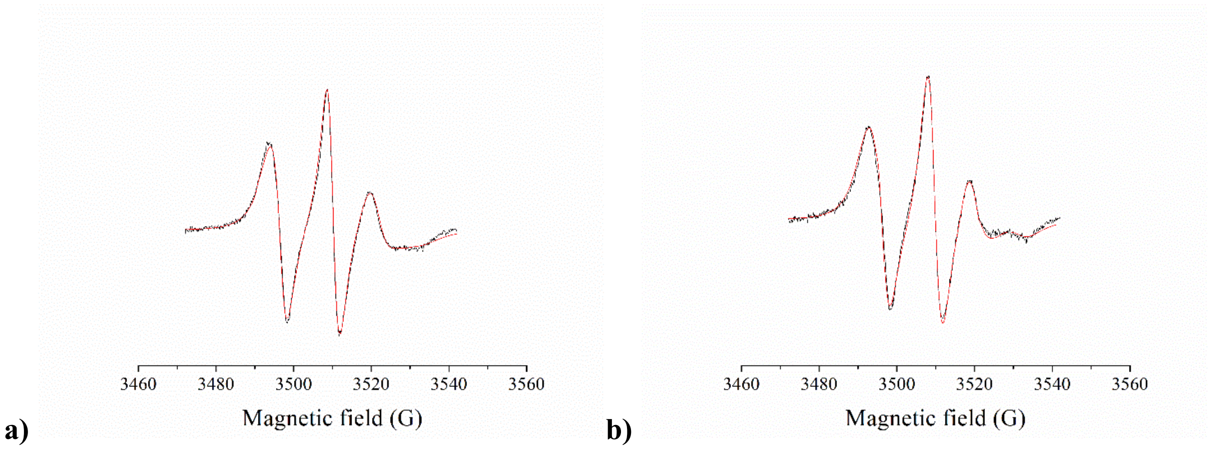

Figure S7 Comparison between experimental (black lines) and computed (red lines) EPR spectra of 14-PCSL in (**a**) chol/POPC/22:6-22:6PC with $x_{22:6\text{-}22:6PC}$=0.5 and (**b**) chol/POPC/18:0-22:6PC with $x_{18:0\text{-}22:6PC}$=0.5 systems.

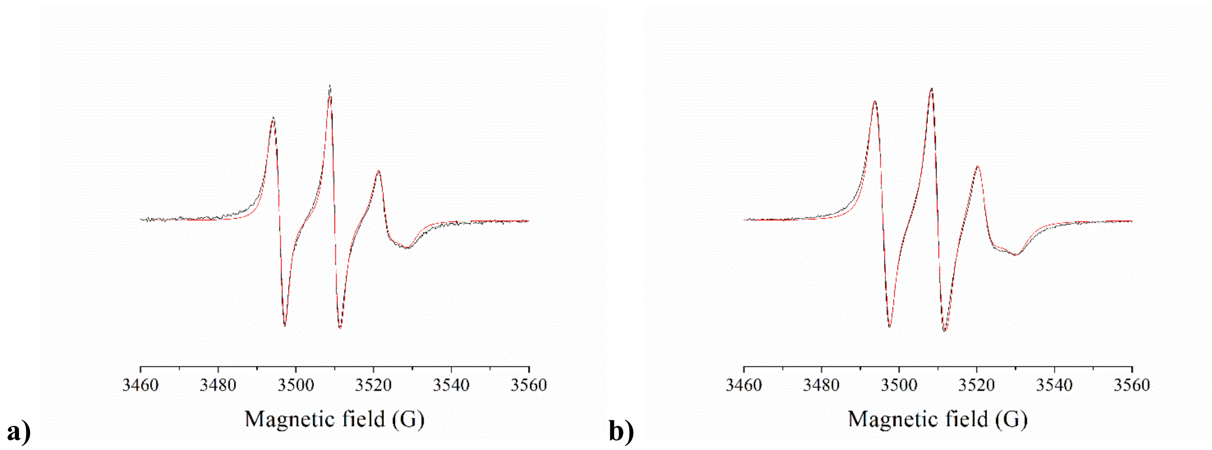

Figure S8 Comparison between experimental (black lines) and computed (red lines) EPR spectra of CNO in (**a**) chol/22:6-22:6PC and (**b**) chol/18:0-22:6PC systems.

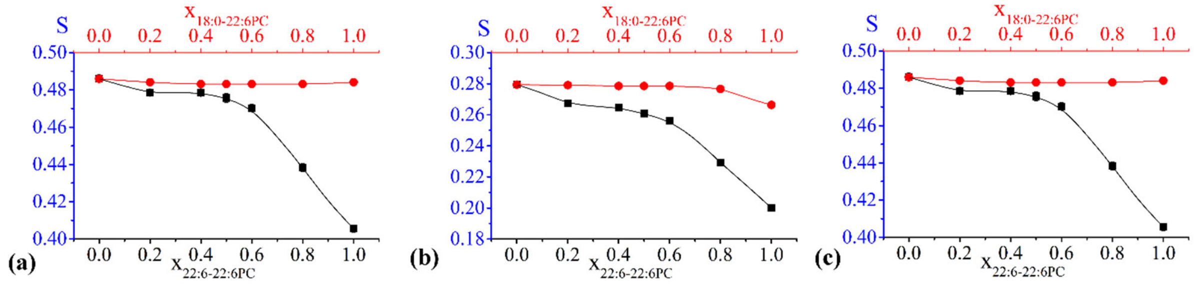

Figure S9 Comparison between S variation as a function of omega-3 phospholipid concentration for chol/POPC/22:6-22:6PC (black) and chol/POPC/18:0-22:6PC (red) systems as obtained by means of (**a**) 5-PCSL, (**b**) 14PCSL, (**c**) CNO.

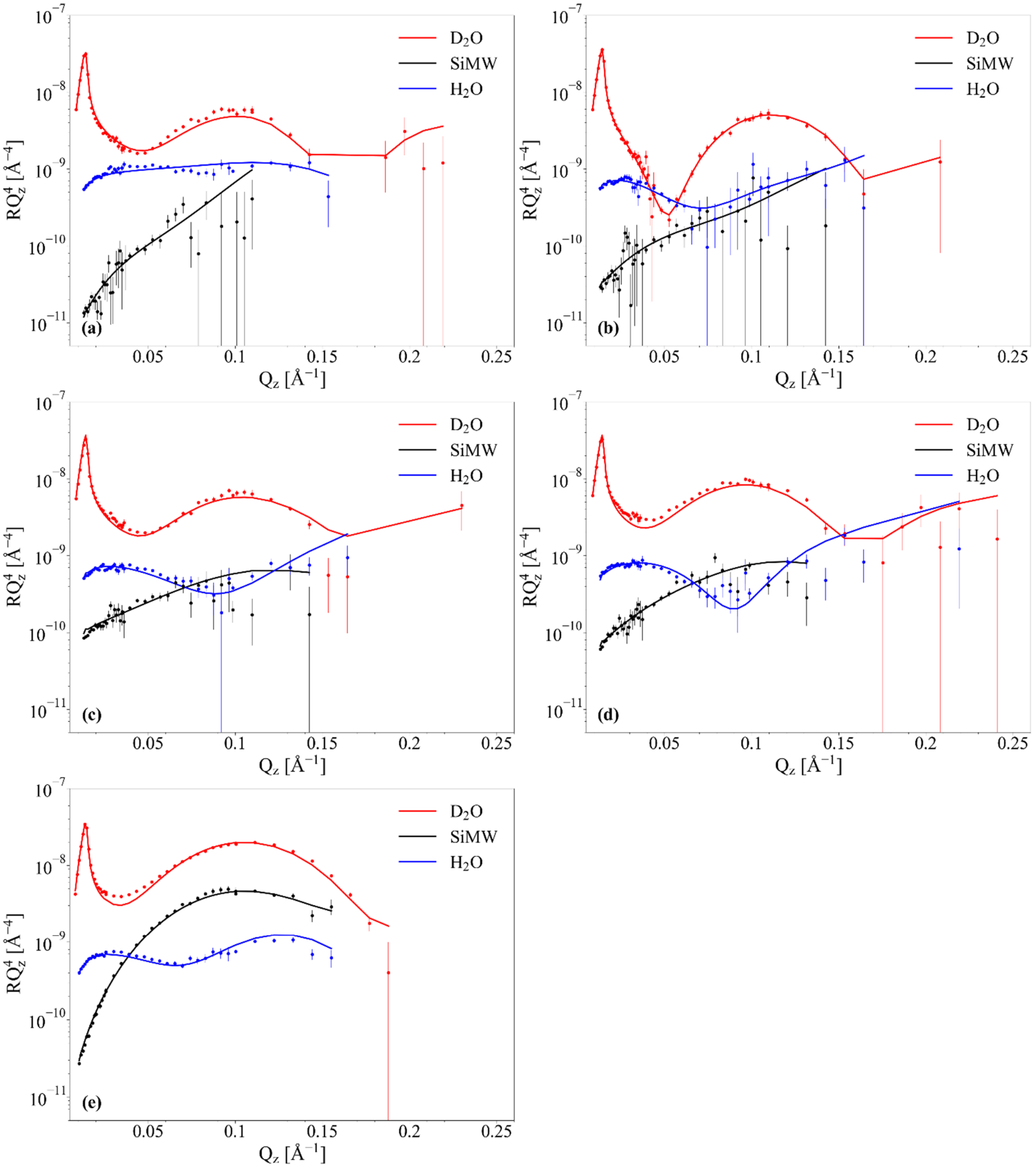

Figure S10 Fresnel representation RQ^4 vs Q of experimental data and best fitting curves in D_2O (red), silicon match water (black) and H_2O (blue) for lipid systems: (**a**) chol/POPC, (**b**) chol/POPC/22:6-22:6PC $x_{22:6\text{-}22:6PC} = 0.2$, (**c**) chol/POPC/22:6-22:6PC $x_{22:6\text{-}22:6PC} = 0.8$, (**d**) chol/POPC/18:0-22:6PC $x_{18:0\text{-}22:6PC} = 0.4$, (**e**) POPC/18:0-22:6PC.

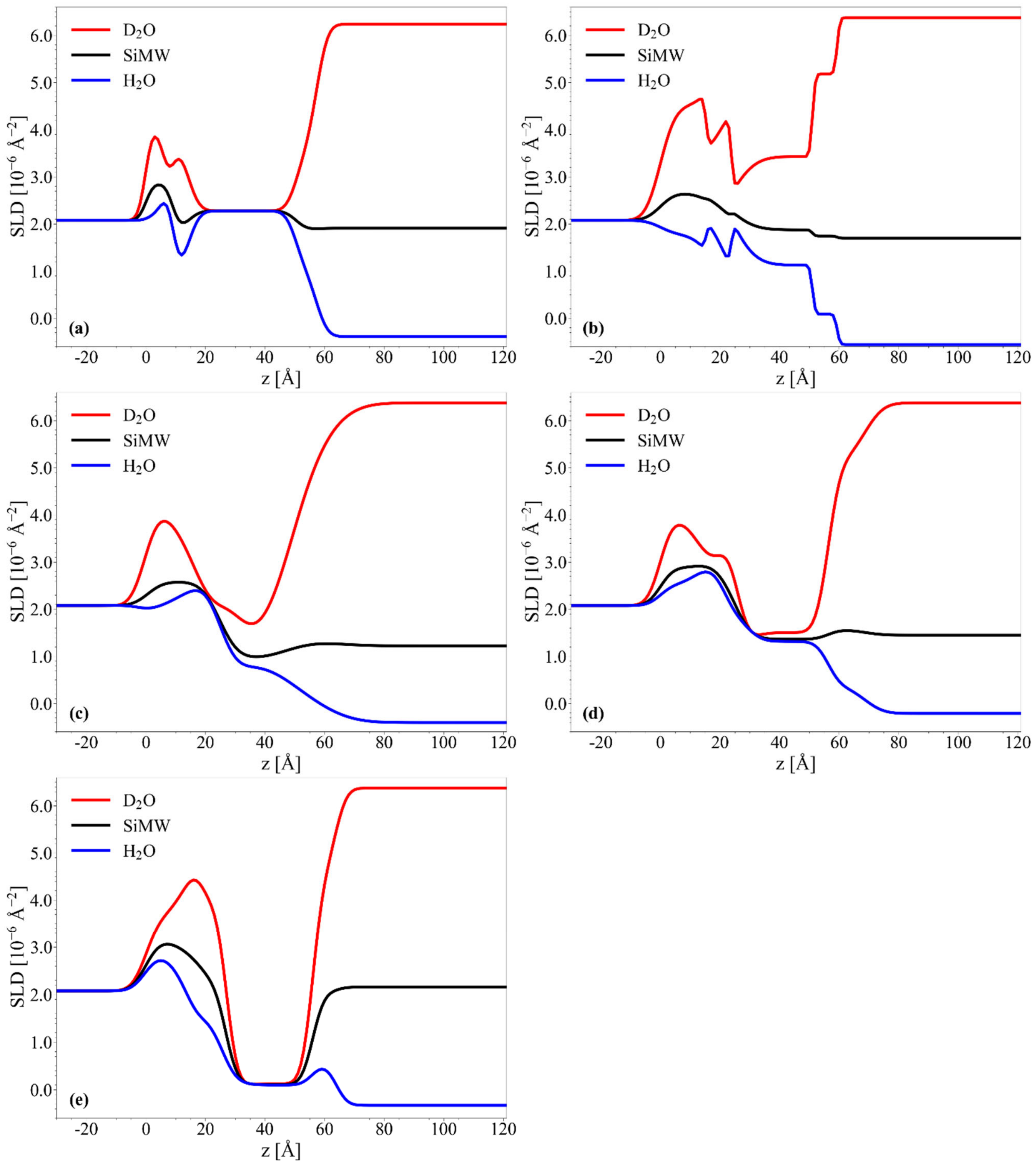

Figure S11 SLD profiles corresponding to best fit of Neutron Reflectivity data in D_2O (red), silicon match water (black) and H_2O (blue) for lipid systems: (**a**) chol/POPC, (**b**) chol/POPC/22:6-22:6PC $x_{22:6\text{-}22:6PC} = 0.2$, (**c**) chol/POPC/22:6-22:6PC $x_{22:6\text{-}22:6PC} = 0.8$, (**d**) chol/POPC/18:0-22:6PC $x_{18:0\text{-}22:6PC} = 0.4$, (**e**) POPC/18:0-22:6PC.

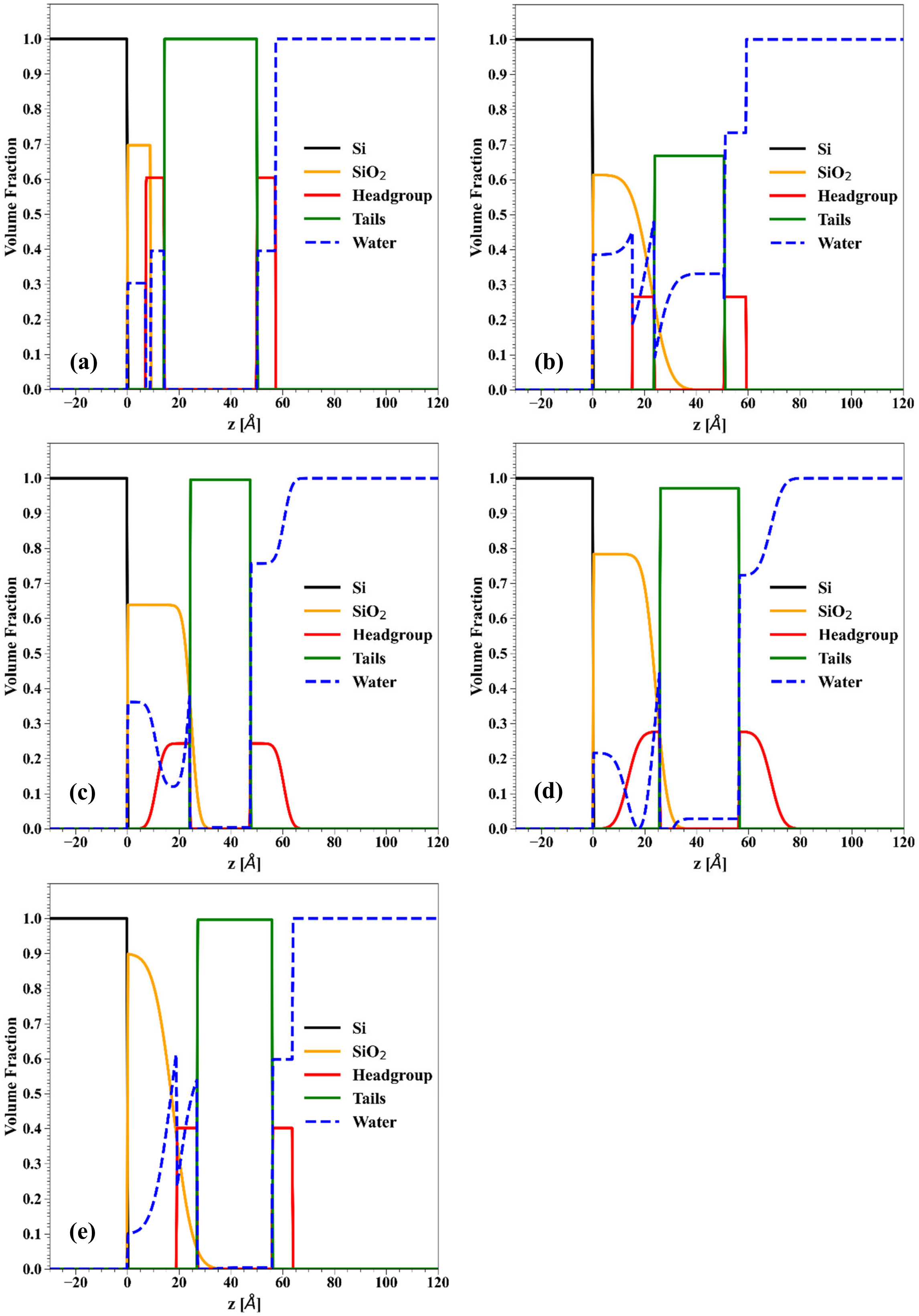

Figure S12 Convolved volume fraction distribution profiles for (**a**) chol/POPC, (**b**) chol/POPC/22:6-22:6PC $x_{22:6\text{-}22:6PC}$ = 0.2, (**c**) chol/POPC/22:6-22:6PC $x_{22:6\text{-}22:6PC}$ = 0.8, (**d**) chol/POPC/18:0-22:6PC $x_{18:0\text{-}22:6PC}$ = 0.4, (**e**) POPC/18:0-22:6PC.

[1] M. Kotlarchyk, S.M. Ritzau, Paracrystal Model of the High-Temperature Lamellar Phase of a Ternary Microemulsion System, J Appl Crystallogr 24 (1991) 753-758, Doi 10.1107/S0021889890012213.
